# Causal relationships between mitochondrial proteins and different pathological types of lung cancer: a bidirectional mendelian randomization study

**DOI:** 10.3389/fgene.2024.1335223

**Published:** 2024-03-26

**Authors:** Tanao Ji, Yue Lv, Meiqun Liu, Yujie Han, Baochang Yuan, Jun Gu

**Affiliations:** ^1^ Department of General Practice, Affiliated Hospital of Nantong University, Medical School of Nantong University, Nantong, China; ^2^ Department of Hematology, Affiliated Hospital of Nantong University, Medical School of Nantong University, Nantong, China; ^3^ Department of Electrocardioeraphy, Qidong People’s Hospital, Qidong Liver Cancer Institute, Affiliated Qidong Hospital of Nantong University, Nantong, China; ^4^ Department of Pulmonary and Critical Care Medicine, Affiliated Hospital of Nantong University, Medical School of Nantong University, Nantong Key Laboratory of Respiratory, Nantong, China

**Keywords:** lung cancer, mitochondrial protein, Mendelian randomization, causal relationship, european

## Abstract

An increasing number of studies point to an association between mitochondrial proteins (MPs) and lung cancer (LC). However, the causal relationship between MPs and LC remains unclear. Consequently, our study employed a bidirectional Mendelian randomization (MR) analysis to explore the causal association between MPs and different pathological types of LC. A two-sample MR study was performed using the genome-wide association study (GWAS) data publicly available. We applied the primary inverse variance weighted (IVW) method along with additional MR methods to validate the causality between MPs and different pathological types of LC. To ensure the robustness of our findings, sensitivity analyses were employed. Moreover, we performed a bi-directional MR analysis to determine the direction of the causal association. We identified a total of seven MPs had significant causal relationships on overall LC, lung squamous cell carcinoma (LUSC), and small cell lung carcinoma (SCLC). We found two MPs had significant associations with overall LC, four MPs had significant associations with LUSC, and four MPs had significant associations with SCLC. Additionally, an MP was found to have a nominal relationship with LUSC. Moreover, no causality was found between MPs and lung adenocarcinoma (LUAD). Bidirectional MR showed no reverse effect between identified MPs and different pathological types of LC. In general, our findings of this MR study suggest causal associations of specific MPs with overall LC, LUSC, and SCLC. However, no such causality was found in LUAD.

## 1 Introduction

Cancer stands as one of the primary causes of global mortality. On a worldwide scale, in 2020, it is estimated that approximately 2.2 million new cases of lung cancer (LC) and nearly 1.8 million LC deaths occurred. Meanwhile, LC ranked as the most commonly diagnosed cancer among males and the third most frequently diagnosed cancer among females (Sung et al., 2021). The incidence of new LC cases is projected to rise until 2035 in most countries, which causes a substantial global public health challenge (Luo et al., 2023). The majority of LC patients are diagnosed at an advanced stage of the disease, resulting in a 5-year survival rate of less than 20% (Osuoha et al., 2018; Bade and Dela Cruz, 2020). Hence, it is crucial to identify modifiable protective or risk factors to prevent the occurrence and progression of LC. Smoking is the most established and well-acknowledged risk factor for LC (Leiter et al., 2023). However, as smoking prevalence decreases and the number of LC cases in nonsmokers rises, it becomes increasingly important to investigate a better understanding of LC development (Bade and Dela Cruz, 2020). As a result, further research is gradually focusing on the other risk factors of LC, encompassing environmental exposures, lifestyle, gender, and genetics (Schabath and Cote, 2019).

Mitochondria serve as the central command for cellular metabolism, maintaining equilibrium and stress responses, playing a pivotal role in regulating processes like cell growth, division, differentiation, and apoptosis (Anderson et al., 2019). Previous studies have unveiled an unforeseen complexity and versatility in mitochondrial activities, combining mitochondrial energetics with protein biogenesis, metabolic pathways, and apoptosis (Pfanner et al., 2019). Moreover, recent studies based on proteomics indicated the remarkable importance of retaining mitochondrial proteostasis in guaranteeing the correct function of mitochondria (Wachoski-Dark et al., 2022). Encoded by both nuclear and mitochondrial DNA, mitochondrial proteins (MPs) are susceptible to errors during folding and assembly on account of oxidative stress and post-translational modifications (Stefani, 2004; Santo-Domingo and Demaurex, 2012). This may result in mitochondrial dysfunction, leading to an increase in reactive oxygen species (ROS) with tumor-promoting effect (Bandy and Davison, 1990). Mitochondrial protein quality control (MPQC) employs various pathways and regulators to maintain the quality and quantity of MPs. Dysregulated MPQC results in proteotoxicity and malfunctioning mitochondria, contributing to a range of human diseases, including cancer. Numerous studies have connected the dysfunction of MPQC in the etiology and pathogenesis of multiple types of cancer, including LC (Wallace, 2012; Friedlander et al., 2021). However, due to various objective factors, including technological and methodological constraints, the majority of existing research findings about MPQC rely on the animal or cellular experiments which can be influenced by multiple variables (Friedlander et al., 2021). In summary, the causality of the relationships between MPs and LC, as well as the direction of these causal connections, remains unclear. Therefore, it is crucial to investigate if MPs contribute to the onset of LC or just outcomes of shared risk factors.

Mendelian randomization (MR) analysis is a widely used method for establishing the causal relationship between exposure factors and outcomes, with the fundamental principle of employing genetic variations as instrumental variables (IVs) to model and evaluate the causality (Sanderson, 2021). The MR approach parallels the design of a randomized controlled trial (RCT) on account of parental alleles being randomly distributed to offspring during gamete formation in Mendel’s law (Emdin et al., 2017). Furthermore, the results of MR studies are more robust against residual confoundings and the bias of reverse causal effects because the genetic variations are randomly assigned during meiosis and are not linked to environmental factors (Boehm and Zhou, 2022).

In our study, we aimed to apply a comprehensive two-sample MR analysis to determine the causal effect between MPs and LC and its various pathological types. By means of employing a bidirectional MR analysis, we could investigate the causality of MPs on LC risk and also determine if LC had a causal effect on MPs. From this foundation, we aimed to elucidate the influence between MPs and different pathological types of LC, ultimately aiding in developing innovative treatment options for LC.

## 2 Methods

### 2.1 Study design

**FIGURE 1:**
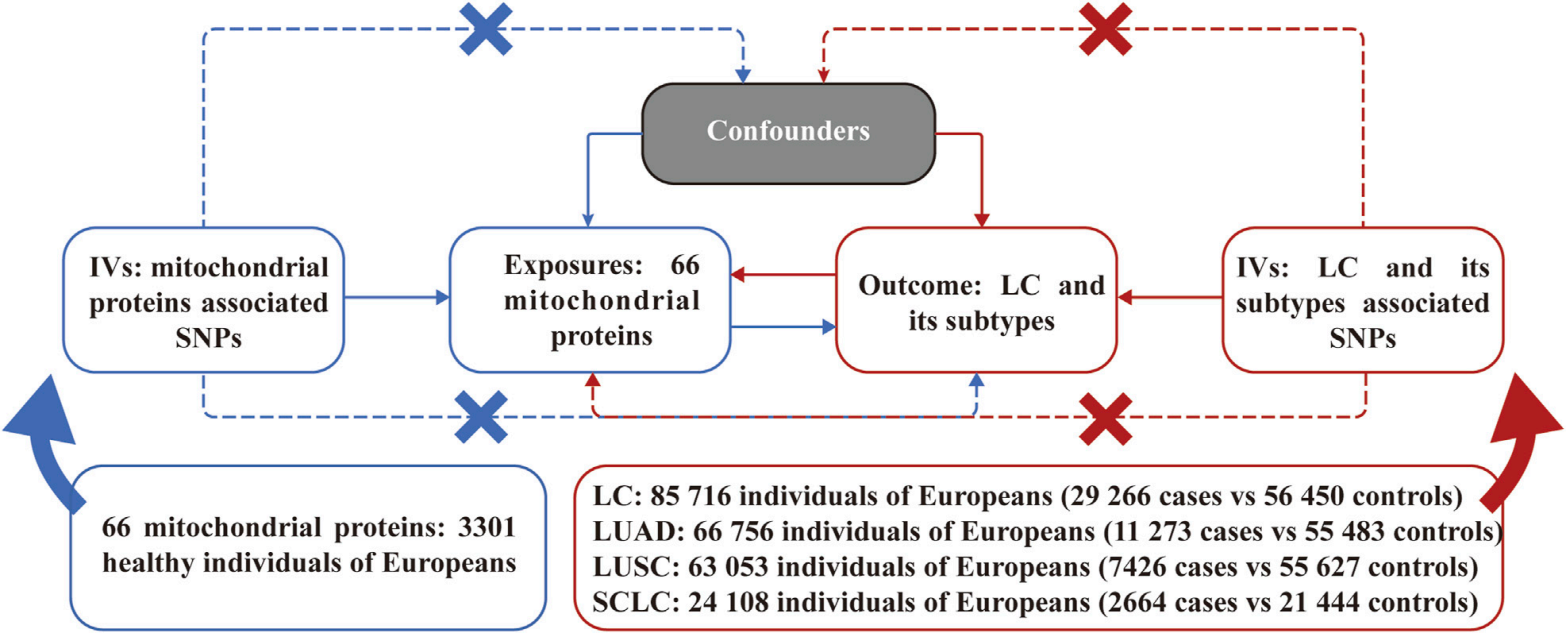
Assumptions and study design of the bidirectional Mendelian randomization study of the causal relationships between 66 mitochondrial proteins and different pathological types of lung cancer. LC, lung cancer; LUAD, lung adenocarcinoma; LUSC, lung squamous cell carcinoma; SCLC, small cell lung carcinoma; SNPs, single nucleotide polymorphisms.

Figure 1 illustrates an overview of the bidirectional MR analyses employed in our study. All IVs selected were guided by three principal assumptions of MR studies. Namely, IVs must demonstrate a strong association with the exposure; IVs impact the outcome solely through the exposure; IVs should not exhibit any association with confounding factors in the relationship between exposure and outcome.

### 2.2 Genome-wide association study (GWAS) sources

The GWAS data for MPs were sourced from a GWAS study involving a total sample size of 3,301 healthy participants of European descent (Sun et al., 2018). A total of 66 mitochondrial proteins (due to limited data availability) were enrolled in the subsequent MR analysis. The GWAS data for LC were derived from a large-scale GWAS study involving 85,716 individuals with 29,266 cases and 56,450 controls, while the GWAS study ulteriorly categorized LC into specific pathological types as lung adenocarcinoma (LUAD), lung squamous cell carcinoma (LUSC), and small cell lung carcinoma (SCLC) (McKay et al., 2017). All detailed information on GWAS data for MR analyses is presented in Figure 1. The original GWAS obtained approval from their respective institutions, and all data used for this study are publicly available. Therefore, no additional ethical approval was required.

### 2.3 Acquisition of IVs

Due to the restricted pool of accessible SNPs, we opted for SNPs with a cutoff of *p* < 1e-5. Then genetic instruments were excluded on a linkage disequilibrium (LD) threshold of r2 < 0.001 and a window size = 10,000 kb. To assess the statistical strength of each SNP, the F statistics were also calculated, and the SNPs with F statistic <10 were eliminated for weak strength (Brion et al., 2013). We further excluded IVs that exhibited associations with potential confounding traits according to PhenoScanner (http://www.phenoscanner.medschl.cam.ac.uk/).

### 2.4 MR analysis

As for the two-sample analyses, we conducted the inverse variance-weighted (IVW) method as the primary approach for examining the bidirectional causal relationships between MPs and different pathological types of LC. Additionally, three complementary MR approaches were employed, including MR-Egger, weighted median (WM), and MR-Pleiotropy residual sum and outlier (MR-PRESSO), to sustain the findings derived from the IVM method. *p*-values were adjusted for false discovery rate (FDR) method, and Adjusted *p*-values (adj. *P*) < 0.05 were considered statistically significant. Also, *p*-values <0.05 were considered nominally significant.

### 2.5 Sensitivity analysis

Given that the IVW method could be biased by pleiotropic IVs, sensitivity analyses were employed to address the pleiotropic effects in the causal estimates. To assess potential heterogeneity, Cochrane’s Q test was applied. In cases where heterogeneity was detected *p* < 0.05, a random-effects IVW analysis was performed to account for the measured heterogeneity. Additionally, the intercept of MR-Egger and MR-PRESSO global test were adopted to estimate the presence of horizontal pleio8tropy in the genetic variants (*p* < 0.05 indicated potential horizontal pleiotropy) while MR-PRESSO global test demonstrated a greater level of accuracy and assistance compared to MR-Egger in identifying horizontal pleiotropy. Furthermore, a leave-one-out analysis was conducted to determine whether the results were actuated by individual variants. We conducted all our MR analyses using the R software (version 4.3.1).

## 3 Results

### 3.1 Acquisition of IVs

After filtering for SNPs with LD, significantly linked to potential confounders (lung function and chronic obstructive pulmonary disease), and other LC-associated traits, a total of 125 SNPs were enrolled as IVs for the ensuing MR analyses, with the F statistics for each SNP being >10, demonstrating the absence of instrument bias (Supplementary Table S1).

### 3.2 Causal effects of MPs on LC

**FIGURE 2:**
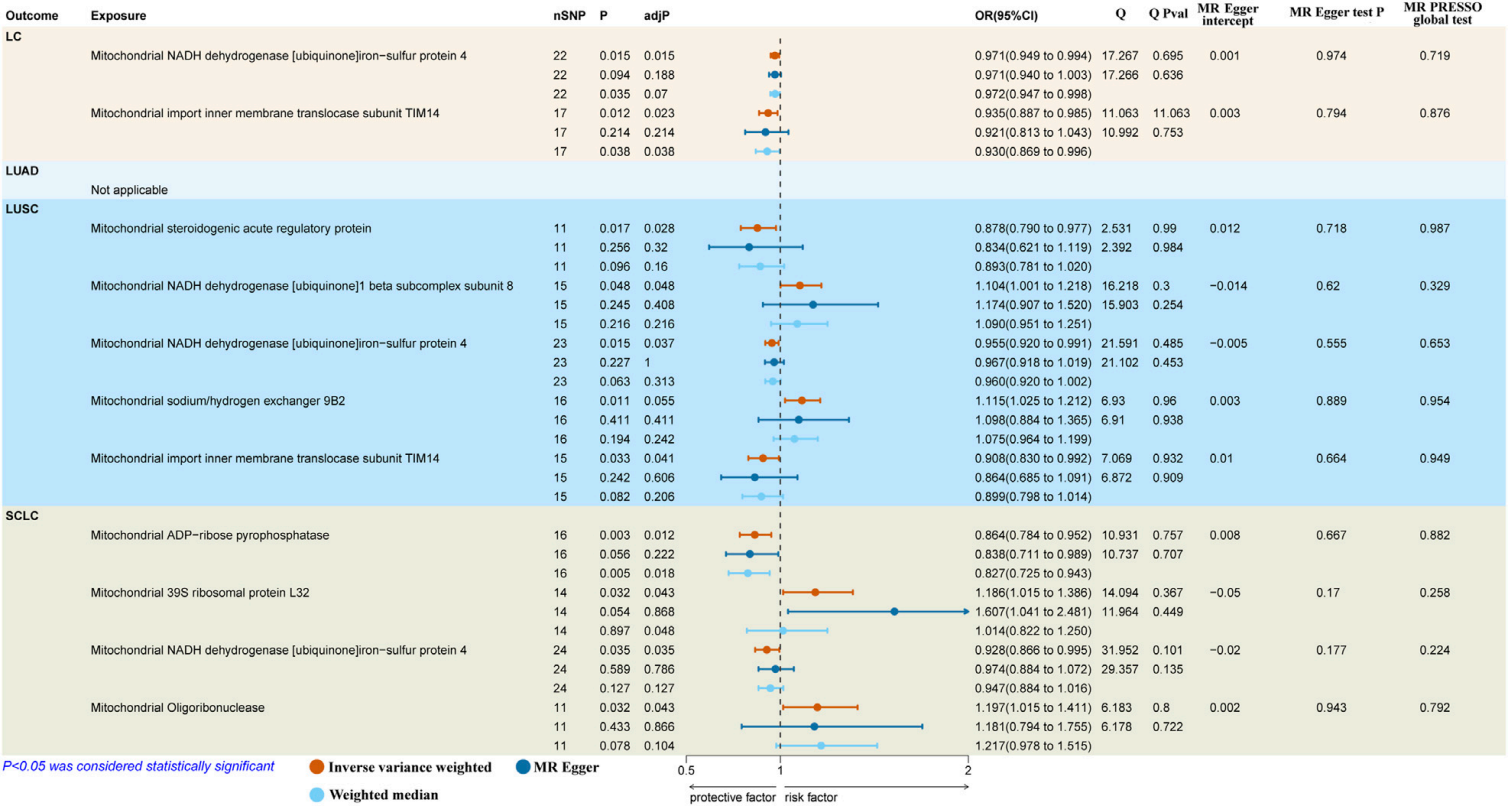
Causal effects of mitochondrial proteins on different pathological types of LC. LC, lung cancer; LUSC, lung squamous cell carcinoma; SCLC, small cell lung carcinoma; SNPs, single nucleotide polymorphisms; MR, Mendelian randomization; MR-PRESSO, MR-pleiotropy residual sum and outlier.

The results reported that both mitochondrial NADH dehydrogenase [ubiquinone]iron-sulfur protein 4 (Ndufs4) (IVW: OR = 0.971, 95% CI: 0.949–0.994, *p* = 0.015, adj. *p* = 0.015) and mitochondrial import inner membrane translocase subunit TIM14 (TIMM14/DNAJC19) (IVW: OR = 0.935, 95% CI: 0.887–0.985, *p* = 0.012, adj. *p* = 0.023) had a protective causal effect on overall LC (Figure 2). However, we observed no genetic predisposition to MPs demonstrated a causal relationship on LUAD (Supplementary Table S2).

As to LUSC, the findings suggested that mitochondrial steroidogenic acute regulatory protein (StAR) (IVW: OR = 0.878, 95% CI 0.790–0.977, *p* = 0.017, adj. *p* = 0.028), mitochondrial Ndufs4 (IVW: OR = 0.955, 95% CI 0.920–0.991, *p* = 0.015; WM: OR = 0.960, 95% CI 0.920–1.002, *p* = 0.063, adj. *p* = 0.037) and mitochondrial DNAJC19 (IVW: OR = 0.908, 95% CI 0.830–0.992, *p* = 0.033, adj. *p* = 0.041) had protective causal effects on LUSC. In contrast, mitochondrial NADH dehydrogenase [ubiquinone]1 beta subcomplex subunit 8 (NDUFB8) (IVW: OR = 1.104, 95% CI 1.001–1.218, *p* = 0.048, adj. *p* = 0.048) indicated pathogenic causal impacts on LUSC. Besides, mitochondrial sodium/hydrogen exchanger 9B2 (SLC9B2) (IVW: OR = 1.115, 95% CI 1.025–1.212, *p* = 0.011, adj. *p* = 0.055) showed a suggestive casual association with the higher risk of LUSC (Figure 2).

In terms of SCLC, we discovered two protective factors, including mitochondrial ADP-ribose pyrophosphatase (NUDT9) (IVW: OR = 0.864, 95% CI 0.784–0.952, *p* = 0.003, adj. *p* = 0.012) and mitochondrial Ndufs4 (IVW: OR = 0.928, 95% CI 0.866–0.995, *p* = 0.035, adj. *p* = 0.048) and two risk factors, including mitochondrial 39S ribosomal protein L32 (MRPL32) (IVW: OR = 1.186, 95% CI 1.015–1.386, *p* = 0.032, adj. *p* = 0.043) and mitochondrial oligoribonuclease (REXO2) (IVW: OR = 1.197, 95% CI 1.015–1.411, *p* = 0.032, adj. *p* = 0.043) were causal associated with SCLC (Figure 2).

### 3.3 Sensitivity analysis

The scatter plot showed that the causal estimates derived by the MR-Egger regression and weighted median approach were consistent in both dimension and direction with IVW method (Supplementary Figure S1). The findings of Cochrane’s Q test indicated no significant heterogeneity (*p* > 0.05). The results revealed that the MR-Egger regression did not identify any pleiotropic effects for MPs (all *p* > 0.05). Additionally, the MR-PRESSO global test detected neither horizontal pleiotropic effects nor outlier SNPs (all *p* > 0.05). Moreover, the leave-one-out analysis validated that no individual SNP solely drove the causality between MPs and different pathological types of LC (Supplementary Figure S2).

### 3.4 Bidirectional causal associations between identified MPs and LC

To assess any reverse causality between identified MPs and different pathological types of LC, we considered overall LC and its subtypes as the exposure and identified MPs as the outcome. After screening, we employed 119 SNPs associated with different pathological types of LC as IVs (Supplementary Table S3). Finally, the results indicated no evidence for a reverse causal association between identified MPs and different pathological types of LC (Table 1).

**TABLE 1:** Causal effects of different pathological types of LC on identified mitochondrial proteins.

Exposure	Outcome	Method	nSNP	OR (95%CI)	*p-*value	adj. *p-value*
LC						
	Mitochondrial NADH dehydrogenase [ubiquinone]iron-sulfur protein 4	Inverse variance weighted	50	1.048 (0.933–1.176)	0.430	0.860
		MR Egger	50	1.083 (0.824–1.424)	0.570	0.570
		Weighted median	50	0.981 (0.831–1.157)	0.816	1
	Mitochondrial import inner membrane translocase subunit TIM14	Inverse variance weighted	50	1.034 (0.897–1.191)	0.649	0.649
		MR Egger	50	1.193 (0.856–1.664)	0.303	0.606
		Weighted median	50	1.012 (0.851–1.204)	0.894	0.894
LUSC						
	Mitochondrial steroidogenic acute regulatory protein	Inverse variance weighted	39	0.952 (0.879–1.031)	0.226	0.377
		MR Egger	39	1.121 (0.949–1.326)	0.188	0.470
		Weighted median	39	0.991 (0.879–1.117)	0.881	0.881
	Mitochondrial NADH dehydrogenase [ubiquinone]1 beta subcomplex subunit 8	Inverse variance weighted	39	0.995 (0.916–1.081)	0.911	0.911
		MR Egger	39	0.971 (0.814–1.159)	0.747	0.747
		Weighted median	39	1.030 (0.914–1.161)	0.626	1.000
	Mitochondrial NADH dehydrogenase [ubiquinone]iron-sulfur protein 4	Inverse variance weighted	39	0.993 (0.907–1.086)	0.872	1.000
		MR Egger	39	0.889 (0.736–1.072)	0.226	0.377
		Weighted median	39	1.023 (0.909–1.151)	0.706	0.883
	Mitochondrial sodium/hydrogen exchanger 9B2	Inverse variance weighted	39	0.948 (0.876–1.027)	0.191	0.478
		MR Egger	39	0.834 (0.706–0.986)	0.041	0.205
		Weighted median	39	0.967 (0.858–1.089)	0.579	1.000
	Mitochondrial import inner membrane translocase subunit TIM14	Inverse variance weighted	39	1.073 (0.987–1.167)	0.098	0.490
		MR Egger	39	1.093 (0.915–1.306)	0.333	0.416
		Weighted median	39	1.082 (0.964–1.213)	0.180	0.900
SCLC						
	Mitochondrial ADP-ribose pyrophosphatase	Inverse variance weighted	30	1.004 (0.945–1.067)	0.902	0.902
		MR Egger	30	1.026 (0.904–1.165)	0.693	0.924
		Weighted median	30	0.987 (0.911–1.070)	0.757	1.000
	Mitochondrial 39S ribosomal protein L32	Inverse variance weighted	30	0.968 (0.911–1.029)	0.298	0.596
		MR Egger	30	0.905 (0.797–1.027)	0.134	0.536
		Weighted median	30	0.940 (0.869–1.017)	0.123	0.492
	Mitochondrial NADH dehydrogenase [ubiquinone]iron-sulfur protein 4	Inverse variance weighted	30	1.057 (0.995–1.123)	0.074	0.296
		MR Egger	30	1.033 (0.910–1.173)	0.619	1.000
		Weighted median	30	1.043 (0.957–1.137)	0.334	0.668
	Mitochondrial Oligoribonuclease	Inverse variance weighted	30	0.995 (0.936–1.057)	0.869	1.000
		MR Egger	30	0.975 (0.858–1.109)	0.708	0.708
		Weighted median	30	1.005 (0.922–1.096)	0.901	0.901

LC, lung cancer. LUAD, lung adenocarcinoma. LUSC, lung squamous cell carcinoma. SCLC, small cell lung carcinoma. SNPs, single nucleotide polymorphisms. MR, Mendelian randomization. adj. *p*-values, Adjusted *p*-values.

## 4 Discussion

To our knowledge, this is the inaugural investigation of the causality between mitochondrial proteins and LC using open-access genetic databases. We employed bidirectional MR analyses to determine the causality between 66 MPs and different pathological types of LC, which enabled us to evaluate the upstream and downstream in the disease progression while avoiding reverse causation. We further ensured the robustness of our MR analyses against pleiotropic influences by implementing a variety of MR approaches, including MR-Egger and MR-PRESSO, to validate our findings. Our results indicated that a total of eight MPs may be potential protective contributors or potential risk factors to the development of overall LC, LUSC, and SCLC, whereas no such causal effect was observed in the case of LUAD. Furthermore, we found no evidence for a reverse causal effect between identified MPs and different pathological types of LC.

The majority of reactive oxygen species (ROS) within cells are produced from the mitochondrial respiratory chain. An overabundance of ROS can result in oxidative stress, causing oxidative harm to proteins and alterations in MP expression (Rabilloud et al., 2001; Poyton et al., 2009). Numerous studies have demonstrated that increased levels of ROS are correlated with the formation and advancement of LC (Weinberg et al., 2010; Jiang et al., 2022). Additionally, examining variations in the mitochondrial proteome is deemed to be an effective method to gauge the degree of mitochondrial damage under oxidative stress conditions (Gibson, 2005). Due to the absence of protective histones and a restricted range of DNA repair mechanisms, mitochondrial DNA (mtDNA) is highly susceptible to oxidative damage (Richter et al., 1988). Instability in mtDNA has been observed in several cancers, including LC (Chatterjee et al., 2006). An observational study suggested that compared to patients without LC, mutation rates in mtDNA were significantly increased in exhaled breath condensate in patients with LC (Yang Ai et al., 2013). After smoking, exposure to radon is the second leading cause of LC (Lorenzo-González et al., 2019). A study found that in radon-induced LC patients, the concentration of cell free mtDNA was significantly increased compared to other participants in the study (Bulgakova et al., 2022). Although these studies suggested a relationship between MPs and LC to some degree, the causal links remain unclear, and the direct association between them still lacks substantial research backing. Our research offered evidence supporting causal associations of MPs with LC and its subtypes by deducing the causality through genetic prediction using MR, which could also mitigate confounders effectively.

The result showed two protective factors in overall LC. Ndufs4 encodes mitochondrial complex I protein (Karamanlidis et al., 2013), while a study revealed deficiency in complex I led to elevated levels of mitochondrial ROS in macrophages in mouse models with myeloid-specific deletion of Ndufs4 (Cai et al., 2023). A similar result of Ndufs4 was also found in LUSC and SCLC, which indicated that Ndufs4 may be a vital protective factor in the development of LC. DNAJC19 plays a crucial role in preserving mitochondrial integrity, and the mutation in DNAJC19 could induce the occurrence of dilated cardiomyopathy and ataxia syndrome (Davey et al., 2006). Paradoxically, the expression of DNAJC19 was increased in NSCLC tissues compared to noncancerous adjacent tissues (Zhou et al., 2021). This conflictive result could be attributed to pathogenic variants in DNAJC19, which can lead to damage to mitochondrial function (Wachoski-Dark et al., 2022). Our finding also indicated the protective causal effect of DNAJC19 on LUSC, which further substantiates that DNAJC19 has a pivotal protective effect against LC.

In terms of LUSC, our study revealed four probable and one possible MPs with causal links, including protective factors of StAR, Ndufs4, and DNAJC19 and risk factors of NDUFB8 and SLC9B2. StAR governs the crucial step that limits the rate of steroid biosynthesis, playing a vital role in the regulation of steroid hormones (Manna et al., 2009). ROS impairs mitochondria, leading to reduced StAR expression and steroidogenesis across various steroid-producing cells. At the same time, hormone deficiencies are considered a primary driver of human aging, which is related to the onset of various tumors (Manna et al., 2016; Jackaman et al., 2017). The selective nitration of NDUFB8 results in the disintegration of mitochondrial supercomplexes, causing the impairment of complex I activity and mitochondrial function. The activity of Complex I is recognized as a crucial factor in controlling mitochondrial respiration. Besides, nitration of NDUFB8 may represent a crucial mechanism in inflammatory conditions, which is a crucial component in the advancement of tumors (Coussens and Werb, 2002; Quintero et al., 2006; Davis et al., 2010). SLC9B2 is a sodium/hydrogen antiporter (Chintapalli et al., 2015). However, our understanding of the precise molecular functions of SLC9B2 remains limited. A previous study suggested the increased expression of SLC9B2 had a positive relationship with Autosomal-dominant polycystic kidney disease (Chapman et al., 2015). The expression level of SLC9B2 was identified significantly upregulated in Crohn’s disease (Ye et al., 2022). We speculate that the inflammation may be one of the reasons for its role as a risk factor for LUSC.

For SCLC, the results identified that NUDT9 and Ndufs4 presented protective causal effects, and MRPL32 and REXO2 showed pathogenic causal effects. Adenosine diphosphate ribose (ADPR) interacts with NUDT9 homology to activate transient receptor potential melastatin 2 (TRPM2) channel (Miller and Cheung, 2016), while the decreased level of TRPM2 was considered to enhance tumor potential metastasis (Gershkovitz et al., 2018). SCLC is well known as a highly aggressive disease, thus this mechanism may explain the association between the protective factor NUDT9 and SCLC. As to risk factors, the current understanding of MRPL32 and REXO2 is limited. Prior studies demonstrated that suppressing MRPL32 could reduce oxygen-glucose deprivation/reperfusion damage (Guan et al., 2020) and that REXO2 was associated with a poorer prognosis in glioma (Wang et al., 2021).

Nevertheless, our study had several constraints. Firstly, increasing the sample size is pivotal for a more accurate determination of the causal relationship between MPs and different pathological types of LC due to the potential biases from the current fairly small MP sample size. Secondly, the participants in GWAS data were predominantly of European populations, which constrained the applicability of our results to other ethnicities and could result in biased conclusions. Finally, our study merely identified causal associations of MPs with LC and its subtypes, further in-depth research is required to clarify the exact mechanisms of the causality.

## 5 Conclusion

In general, we systematically assessed the causality between MPs and different pathological types of LC by performing bidirectional MR analyses. Our study identified a total of seven MPs had significant causal relationships on overall LC, LUSC, and SCLC. Our findings suggested that there were two protective causal associations with LC; two protective causal associations, two causal pathogenic associations, and a nominally protective causal association with LUSC; two protective causal associations and two causal pathogenic associations with SCLC. Additionally, the results demonstrated no MP had a causality link with LUAD, and no evidence supported the reverse causality for identified MPs with LC or its subtypes. This research underscores the causal effects of MPs on the occurrence of LC, suggesting that MPs might be a viable strategy for LC prevention.

## Data Availability

Publicly available datasets were analyzed in this study. This data can be found here: https://www.ebi.ac.uk/gwas/publications/29875488. https://www.ebi.ac.uk/gwas/publications/28604730.
