# Supplementary material for: Causal relationships between mitochondrial proteins and different pathological types of lung cancer: a bidirectional mendelian randomization study
Source: Front Genet. 2024 Mar 26;15:1335223. doi: 10.3389/fgene.2024.1335223 (PMC11002161; doi:10.3389/fgene.2024.1335223)
Supplement: Supplementary file 1 [file Table1.DOCX]

Supplementary Material

Causal relationships between mitochondrial proteins and different pathological types of lung cancer: A bidirectional Mendelian randomization study

**Tanao Ji^1†^, Yue Lv^2†^, Meiqun Liu^3^, Yujie Han^4^, Baochang Yuan^4^, Jun Gu**^4*^

- **Table S1**: Characteristics of GWAS-identified mitochondrial proteins-associated genetic instruments.
- **Table S2**：Causal effects of mitochondrial proteins on lung adenocarcinoma.
- **Table S3**: Characteristics of GWAS-identified different pathological types of LC-associated genetic instruments.
- **Figure S1**: Scatter plot for the associations between mitochondrial proteins against risk of different pathological types of lung cancer. (A) Mitochondrial NADH dehydrogenase [ubiquinone]iron-sulfur protein 4 on LC. (B) Mitochondrial import inner membrane translocase subunit TIM14 on LC. (C) Mitochondrial steroidogenic acute regulatory protein on LUSC. (D) Mitochondrial NADH dehydrogenase [ubiquinone]1 beta subcomplex subunit 8 on LUSC. (E) Mitochondrial NADH dehydrogenase [ubiquinone]iron-sulfur protein 4 on LUSC. (F) Mitochondrial sodium/hydrogen exchanger 9B2 on LUSC. (G) Mitochondrial import inner membrane translocase subunit TIM14 on LUSC. (H) Mitochondrial ADP-ribose pyrophosphatase on SCLC. (I) Mitochondrial 39S ribosomal protein L32 on SCLC. (J) Mitochondrial NADH dehydrogenase [ubiquinone]iron-sulfur protein 4 on SCLC. (K) Mitochondrial Oligoribonuclease on SCLC. LC, lung cancer; LUSC, lung squamous cell carcinoma; SCLC, small cell lung carcinoma; SNPs, single nucleotide polymorphisms; MR, mendelian randomization; MR-PRESSO, MR-pleiotropy residual sum and outlier.
- **Figure S2**: Leave-one-out analysis for the effect of mitochondrial proteins and different pathological types of lung cancer. (A) Mitochondrial NADH dehydrogenase [ubiquinone]iron-sulfur protein 4 on LC. (B) Mitochondrial import inner membrane translocase subunit TIM14 on LC. (C) Mitochondrial steroidogenic acute regulatory protein on LUSC. (D) Mitochondrial NADH dehydrogenase [ubiquinone]1 beta subcomplex subunit 8 on LUSC. (E) Mitochondrial NADH dehydrogenase [ubiquinone]iron-sulfur protein 4 on LUSC. (F) Mitochondrial sodium/hydrogen exchanger 9B2 on LUSC. (G) Mitochondrial import inner membrane translocase subunit TIM14 on LUSC. (H) Mitochondrial ADP-ribose pyrophosphatase on SCLC. (I) Mitochondrial 39S ribosomal protein L32 on SCLC. (J) Mitochondrial NADH dehydrogenase [ubiquinone]iron-sulfur protein 4 on SCLC. (K) Mitochondrial Oligoribonuclease on SCLC. LC, lung cancer; LUSC, lung squamous cell carcinoma; SCLC, small cell lung carcinoma.

| Table S1. Characteristics of GWAS-identified mitochondrial proteins-associated genetic instruments. | | | | | | | | | | |
| --- | --- | --- | --- | --- | --- | --- | --- | --- | --- | --- |
| **Expsure** | **SNP** | **Chromosome** | **Position** | **Effect allele** | **Other allele** | **Effect allele frequency** | **Beta** | **Standard Error** | ***P*-value** | ***F*-statistic** |
| Mitochondrial NADH dehydrogenase [ubiquinone]iron-sulfur protein 4 | rs111558233 | 3 | 151035399 | T | C | 0.16309 | 0.1511 | 0.0334 | 0.00000603 | 20.4537426 |
|  | rs115713928 | 2 | 118611585 | A | G | 0.38194 | -0.1189 | 0.0254 | 0.00000275 | 21.89944291 |
|  | rs11739292 | 5 | 26198575 | T | G | 0.47753 | 0.1146 | 0.0256 | 0.00000776 | 20.02747028 |
|  | rs117823715 | 8 | 130810193 | G | T | 0.03035 | 0.3496 | 0.0768 | 0.00000537 | 20.70890802 |
|  | rs12355543 | 10 | 478185 | G | T | 0.06824 | -0.2329 | 0.0506 | 0.00000407 | 21.17262642 |
|  | rs1329424 | 1 | 196646176 | G | T | 0.61159 | 0.8670 | 0.0205 | 1E-200 | 1787.587314 |
|  | rs150634249 | 5 | 66576729 | A | G | 0.01975 | -0.4320 | 0.0952 | 0.00000575 | 20.57929004 |
|  | rs17020942 | 3 | 28118321 | A | G | 0.02006 | -0.4448 | 0.0957 | 0.00000339 | 21.58949445 |
|  | rs17584783 | 3 | 2895719 | T | C | 0.44904 | 0.1178 | 0.0254 | 0.00000339 | 21.49611312 |
|  | rs2640498 | 1 | 111182544 | G | A | 0.58276 | 0.1236 | 0.0262 | 0.00000229 | 22.24186242 |
|  | rs2782542 | 1 | 61781773 | A | G | 0.2474 | 0.1391 | 0.0306 | 0.00000537 | 20.65133816 |
|  | rs34140990 | 11 | 69674339 | A | C | 0.42058 | 0.1193 | 0.0251 | 0.00000195 | 22.57720808 |
|  | rs4111399 | 9 | 79727521 | C | T | 0.4589 | -0.1143 | 0.025 | 0.0000049 | 20.89051924 |
|  | rs523427 | 1 | 197223447 | G | A | 0.95536 | -0.2753 | 0.0601 | 0.00000457 | 20.97008882 |
|  | rs55843549 | 8 | 92318893 | G | T | 0.58004 | 0.1192 | 0.0251 | 0.00000214 | 22.53937447 |
|  | rs7335075 | 13 | 54732769 | T | C | 0.87156 | 0.1660 | 0.0373 | 0.00000871 | 19.7940792 |
|  | rs74927039 | 6 | 93486616 | A | G | 0.07105 | 0.2289 | 0.0485 | 0.0000024 | 22.26101177 |
|  | rs75034267 | 8 | 13897979 | G | A | 0.01528 | 0.4790 | 0.1008 | 0.00000204 | 22.56767162 |
|  | rs75969601 | 20 | 56768341 | A | C | 0.06086 | 0.2476 | 0.0519 | 0.00000186 | 22.74591208 |
|  | rs7777571 | 7 | 11758271 | G | T | 0.24906 | -0.1422 | 0.0291 | 0.000001 | 23.86437177 |
|  | rs79685806 | 6 | 75923886 | A | C | 0.01496 | 0.4793 | 0.1073 | 0.00000794 | 19.94124206 |
|  | rs8013508 | 14 | 33172438 | A | G | 0.11971 | -0.1804 | 0.0391 | 0.00000398 | 21.27435211 |
|  | rs9877675 | 3 | 162751412 | G | A | 0.39374 | 0.1222 | 0.0255 | 0.00000166 | 22.95085357 |
|  | rs116499586 | 9 | 139077587 | A | G | 0.05167 | -0.2771 | 0.05167 | 0.00000575 | 20.55547052 |
|  | rs143366101 | 2 | 149478159 | T | C | 0.014 | -0.5585 | 0.014 | 0.00000129 | 23.40838914 |
| Mitochondrial import inner membrane translocase subunit TIM14 | rs117929041 | 8 | 101113296 | A | G | 0.02275 | -0.3955 | 0.0819 | 0.00000138 | 23.30568989 |
|  | rs118141449 | 6 | 138619049 | A | G | 0.0066 | 0.7555 | 0.1658 | 0.00000513 | 20.75088751 |
|  | rs1395661 | 4 | 170094120 | G | A | 0.26182 | -0.1331 | 0.0286 | 0.00000331 | 21.64516177 |
|  | rs148562138 | 15 | 95542721 | T | C | 0.01156 | 0.5505 | 0.1234 | 0.00000813 | 19.88937419 |
|  | rs150664277 | 7 | 10822703 | T | G | 0.01668 | 0.4598 | 0.1031 | 0.00000813 | 19.8773001 |
|  | rs17431748 | 8 | 119090347 | G | T | 0.06097 | -0.2476 | 0.0546 | 0.00000575 | 20.55193826 |
|  | rs2275457 | 1 | 9427869 | A | G | 0.81939 | -0.1503 | 0.0321 | 0.00000282 | 21.91011654 |
|  | rs2441488 | 2 | 114299607 | C | T | 0.75264 | -0.1442 | 0.0326 | 0.00000955 | 19.55384246 |
|  | rs35096982 | 17 | 71927301 | A | G | 0.0775 | -0.2119 | 0.0471 | 0.00000676 | 20.22818377 |
|  | rs62054235 | 16 | 80977137 | T | C | 0.03357 | 0.3962 | 0.0714 | 2.88E-08 | 30.77296267 |
|  | rs71545349 | 7 | 141695892 | T | C | 0.06489 | 0.2391 | 0.0498 | 0.00000162 | 23.0376013 |
|  | rs7173933 | 15 | 61835274 | A | G | 0.8094 | -0.1423 | 0.0315 | 0.00000631 | 20.39508332 |
|  | rs7623475 | 3 | 72573754 | T | C | 0.87066 | -0.1819 | 0.0377 | 0.00000145 | 23.26588028 |
|  | rs76492223 | 20 | 38744230 | T | G | 0.06517 | -0.2218 | 0.0494 | 0.00000708 | 20.14679545 |
|  | rs79269008 | 12 | 56614680 | G | A | 0.02205 | -0.3786 | 0.0832 | 0.00000537 | 20.69432941 |
|  | rs79721732 | 7 | 46311668 | C | T | 0.18064 | -0.1463 | 0.0324 | 0.00000646 | 20.37677374 |
|  | rs79914669 | 18 | 10828663 | T | C | 0.03463 | -0.3346 | 0.0686 | 0.00000107 | 23.77608984 |
| Mitochondrial steroidogenic acute regulatory protein | rs10048951 | 3 | 114570637 | A | G | 0.91497 | 0.2099 | 0.044 | 0.00000182 | 22.74344848 |
|  | rs115542122 | 2 | 213048051 | C | T | 0.07406 | 0.2542 | 0.0517 | 0.000000891 | 24.16054894 |
|  | rs116983610 | 6 | 79620375 | C | T | 0.04808 | 0.2776 | 0.0605 | 0.00000447 | 21.04093164 |
|  | rs117374296 | 11 | 87306303 | C | T | 0.0272 | -0.3979 | 0.0775 | 0.000000288 | 26.34397251 |
|  | rs12448062 | 16 | 82622800 | A | G | 0.96839 | -0.3718 | 0.0708 | 0.000000151 | 27.56062403 |
|  | rs12736482 | 1 | 19817942 | A | C | 0.04106 | -0.3019 | 0.0676 | 0.00000794 | 19.93286041 |
|  | rs145112111 | 9 | 76661907 | T | C | 0.01971 | -0.4506 | 0.0944 | 0.00000178 | 22.77063686 |
|  | rs149892573 | 10 | 32684835 | A | G | 0.03571 | -0.3055 | 0.0681 | 0.00000724 | 20.11245088 |
|  | rs1891240 | 1 | 236694594 | G | A | 0.72201 | -0.1443 | 0.0278 | 0.000000209 | 26.92649725 |
|  | rs4638841 | 2 | 33098175 | A | G | 0.1849 | -0.1511 | 0.0319 | 0.00000214 | 22.42251658 |
|  | rs79633151 | 13 | 109852534 | T | C | 0.03386 | 0.3092 | 0.0694 | 0.00000832 | 19.83795135 |
| Mitochondrial NADH dehydrogenase [ubiquinone]1 beta subcomplex subunit 8 | rs114318458 | 1 | 200586126 | T | C | 0.06351 | 0.2537 | 0.0552 | 0.00000427 | 21.11055107 |
|  | rs1353654 | 4 | 28264394 | T | C | 0.1616 | 0.1490 | 0.0336 | 0.00000933 | 19.65308685 |
|  | rs139685367 | 7 | 13883126 | T | C | 0.02296 | -0.4320 | 0.0919 | 0.00000257 | 22.08377238 |
|  | rs140609555 | 1 | 88359214 | C | T | 0.01116 | -0.5646 | 0.1202 | 0.00000263 | 22.05005126 |
|  | rs146972926 | 16 | 68717768 | T | C | 0.04156 | -0.3032 | 0.065 | 0.00000309 | 21.74545363 |
|  | rs148607169 | 14 | 79232085 | T | C | 0.04007 | -0.3155 | 0.0694 | 0.00000537 | 20.65458995 |
|  | rs150769330 | 12 | 14272609 | T | C | 0.03415 | 0.3243 | 0.0716 | 0.00000589 | 20.50239724 |
|  | rs190846883 | 4 | 58924427 | A | G | 0.08926 | -0.2184 | 0.0465 | 0.00000257 | 22.04632237 |
|  | rs2487417 | 6 | 70554416 | G | A | 0.06193 | 0.2454 | 0.0533 | 0.00000427 | 21.1851474 |
|  | rs253946 | 5 | 131330461 | A | G | 0.80894 | 0.1509 | 0.0319 | 0.00000234 | 22.36319778 |
|  | rs607747 | 1 | 23600610 | G | A | 0.70759 | -0.1278 | 0.0285 | 0.00000741 | 20.09596098 |
|  | rs62098057 | 18 | 37997223 | A | G | 0.06 | 0.2353 | 0.0521 | 0.00000617 | 20.38474105 |
|  | rs68186589 | 15 | 61842539 | A | G | 0.18443 | 0.1440 | 0.0321 | 0.00000708 | 20.11183561 |
|  | rs74935952 | 1 | 237576393 | G | A | 0.06246 | -0.2387 | 0.0508 | 0.00000263 | 22.06552194 |
|  | rs75714219 | 14 | 42820980 | G | A | 0.01136 | 0.5703 | 0.1287 | 0.00000933 | 19.62395054 |
| Mitochondrial sodium/hydrogen exchanger 9B2 | rs113674612 | 7 | 128778386 | A | G | 0.07257 | -0.2185 | 0.049 | 0.00000832 | 19.87227158 |
|  | rs118187045 | 17 | 2980532 | G | A | 0.0946 | -0.1919 | 0.0411 | 0.00000302 | 21.78728412 |
|  | rs13285688 | 9 | 6461678 | T | C | 0.04126 | -0.2813 | 0.0628 | 0.00000741 | 20.05196639 |
|  | rs138449860 | 8 | 105738823 | G | T | 0.01047 | 0.5632 | 0.1266 | 0.00000871 | 19.77856014 |
|  | rs144085636 | 5 | 87247694 | A | C | 0.01303 | -0.5010 | 0.113 | 0.00000933 | 19.64515031 |
|  | rs150356321 | 15 | 98463502 | A | G | 0.0199 | -0.4246 | 0.0881 | 0.00000145 | 23.21373135 |
|  | rs17106210 | 1 | 81685352 | G | A | 0.07755 | 0.2092 | 0.0471 | 0.00000891 | 19.71597857 |
|  | rs1966663 | 17 | 73333871 | C | T | 0.78688 | -0.1341 | 0.0299 | 0.00000741 | 20.10258792 |
|  | rs2192594 | 14 | 73335169 | T | C | 0.16659 | -0.1482 | 0.0334 | 0.00000933 | 19.67615635 |
|  | rs2473520 | 6 | 139431060 | T | G | 0.22595 | -0.1393 | 0.0295 | 0.00000229 | 22.28409453 |
|  | rs62125989 | 19 | 49475876 | T | C | 0.31915 | 0.1347 | 0.0275 | 0.000001 | 23.97764881 |
|  | rs7144298 | 14 | 106248399 | A | G | 0.63198 | -0.2577 | 0.0276 | 1.17E-20 | 87.12593748 |
|  | rs73174837 | 3 | 177326538 | C | T | 0.06048 | 0.2306 | 0.0518 | 0.00000832 | 19.80595909 |
|  | rs74629434 | 12 | 24704817 | T | C | 0.04895 | -0.2756 | 0.061 | 0.00000617 | 20.40025273 |
|  | rs7845776 | 8 | 78800795 | G | A | 0.36538 | 0.1131 | 0.0252 | 0.00000708 | 20.13079469 |
|  | rs78502515 | 2 | 67108558 | G | A | 0.08921 | -0.2079 | 0.0429 | 0.00000126 | 23.47097795 |
| Mitochondrial ADP-ribose pyrophosphatase | rs10262052 | 7 | 11485527 | C | T | 0.50282 | 0.1213 | 0.025 | 0.00000126 | 23.5276405 |
|  | rs1028021 | 8 | 139269519 | G | T | 0.45595 | 0.1119 | 0.0245 | 0.00000479 | 20.84801907 |
|  | rs10959466 | 9 | 11022581 | T | C | 0.20008 | -0.1557 | 0.0313 | 0.000000676 | 24.73006975 |
|  | rs116726256 | 2 | 1855118 | C | T | 0.01616 | -0.4525 | 0.0996 | 0.0000055 | 20.62791254 |
|  | rs12522716 | 5 | 78761776 | C | T | 0.12734 | 0.1723 | 0.0377 | 0.00000479 | 20.87491163 |
|  | rs13336405 | 16 | 52044724 | C | T | 0.05834 | -0.2440 | 0.0538 | 0.00000575 | 20.55662875 |
|  | rs138597300 | 7 | 136528586 | C | T | 0.00869 | 0.6440 | 0.1406 | 0.00000468 | 20.96707673 |
|  | rs1801689 | 17 | 64210580 | C | A | 0.03043 | 1.0267 | 0.0694 | 1.38E-49 | 218.7282984 |
|  | rs1872195 | 16 | 85457875 | A | G | 0.63435 | -0.1161 | 0.0256 | 0.00000562 | 20.55518075 |
|  | rs2812171 | 6 | 67188306 | C | A | 0.86978 | -0.1674 | 0.0371 | 0.00000661 | 20.3469763 |
|  | rs28696943 | 4 | 88310135 | G | A | 0.16685 | -0.3571 | 0.0323 | 2.29E-28 | 122.1550559 |
|  | rs4132079 | 3 | 34044246 | A | C | 0.35161 | 0.1196 | 0.026 | 0.00000427 | 21.14717964 |
|  | rs62143196 | 19 | 54320636 | G | A | 0.22404 | 0.1385 | 0.0303 | 0.0000049 | 20.88098979 |
|  | rs71458836 | 12 | 124792451 | A | G | 0.1403 | 0.1725 | 0.0379 | 0.00000525 | 20.70315674 |
|  | rs75394259 | 16 | 71825631 | T | C | 0.0524 | -0.2611 | 0.0558 | 0.00000282 | 21.88175429 |
|  | rs790121 | 3 | 122389364 | G | T | 0.09558 | 0.1990 | 0.0429 | 0.00000347 | 21.504451 |
| Mitochondrial 39S ribosomal protein L32 | rs111823183 | 10 | 99017869 | T | C | 0.04822 | -0.2938 | 0.0639 | 4.36516E-06 | 21.12704016 |
|  | rs11265892 | 9 | 92050828 | A | G | 0.04085 | -0.2782 | 0.0622 | 7.76247E-06 | 19.99264586 |
|  | rs16878004 | 8 | 31255940 | T | C | 0.31122 | -0.1202 | 0.0267 | 6.60693E-06 | 20.25457822 |
|  | rs2042809 | 8 | 82519771 | G | A | 0.21786 | -0.1370 | 0.0306 | 7.58578E-06 | 20.03249636 |
|  | rs34301109 | 16 | 88614310 | C | T | 0.59096 | -0.1226 | 0.0251 | 1.04713E-06 | 23.84351551 |
|  | rs6043363 | 20 | 15542612 | C | T | 0.03963 | -0.2919 | 0.0657 | 8.70964E-06 | 19.72759948 |
|  | rs61866216 | 10 | 68741032 | C | T | 0.24913 | -0.1298 | 0.0294 | 9.77237E-06 | 19.48011495 |
|  | rs61978300 | 14 | 78930598 | T | C | 0.01854 | 0.4494 | 0.0962 | 2.95121E-06 | 21.80985525 |
|  | rs6827019 | 4 | 48506799 | A | G | 0.18189 | -0.1520 | 0.0319 | 1.86209E-06 | 22.69042346 |
|  | rs71520685 | 8 | 128880188 | G | A | 0.04594 | -0.2779 | 0.0599 | 3.54813E-06 | 21.51098216 |
|  | rs75733115 | 18 | 61989983 | T | C | 0.01964 | 0.4296 | 0.0928 | 3.71535E-06 | 21.41753001 |
|  | rs769162 | 1 | 102304233 | A | G | 0.21821 | 0.1423 | 0.0322 | 9.77237E-06 | 19.51797907 |
|  | rs7989007 | 13 | 91937641 | G | A | 0.57556 | 0.1208 | 0.0264 | 4.67735E-06 | 20.92487181 |
|  | rs9946277 | 18 | 35501945 | A | G | 0.27159 | -0.1284 | 0.0283 | 5.62341E-06 | 20.5728267 |
| Mitochondrial Oligoribonuclease | rs10781989 | 16 | 77866815 | A | C | 0.49062 | 0.1133 | 0.0256 | 0.00000977 | 19.57567203 |
|  | rs11025367 | 11 | 20090650 | C | T | 0.14384 | 0.1668 | 0.0361 | 0.0000038 | 21.33607258 |
|  | rs145878096 | 6 | 17368048 | T | C | 0.01712 | -0.4440 | 0.0978 | 0.00000562 | 20.59799849 |
|  | rs148294894 | 14 | 71909426 | T | C | 0.02362 | -0.3895 | 0.0814 | 0.0000017 | 22.8824702 |
|  | rs28508503 | 4 | 66550173 | A | C | 0.05458 | 0.2464 | 0.0551 | 0.00000759 | 19.9854992 |
|  | rs4608937 | 5 | 177510571 | G | A | 0.68678 | 0.1227 | 0.0266 | 0.00000398 | 21.26486566 |
|  | rs4822928 | 22 | 28089737 | A | G | 0.04093 | 0.2902 | 0.0626 | 0.00000363 | 21.47746109 |
|  | rs61900292 | 11 | 110748408 | T | C | 0.47035 | 0.1225 | 0.025 | 0.000001 | 23.99545289 |
|  | rs62315047 | 4 | 109693555 | A | C | 0.04842 | 0.2895 | 0.065 | 0.00000851 | 19.82472693 |
|  | rs6695899 | 1 | 153215661 | T | G | 0.45541 | -0.1152 | 0.0246 | 0.00000288 | 21.91651692 |
|  | rs71566312 | 6 | 100463816 | C | T | 0.03878 | 0.3176 | 0.0661 | 0.00000158 | 23.0725109 |
| F-statistic was calculated using the following formulas: F=R^2 (n-2)⁄(1-R^2) and R^2=2×MAF×(1-MAF)×β^2, where F represents F-statistic, R^2 represents the phenotypic variance explained by a genetic instrument, N is the sample size, β is the estimated genetic association of SNP with the exposure. SNP: single nucleotide polymorphisms. | | | | | | | | | | |

| Table S2. Causal effects of mitochondrial proteins on lung adenocarcinoma. | | | | | | |
| --- | --- | --- | --- | --- | --- | --- |
| **Exposure** | **Exposure.id** | **Outconme** | **Method** | **nSNP** | **OR (95%CI)** | ***P-*value** |
| 39S ribosomal protein L33, mitochondrial | prot-a-1942 | Lung adenocarcinoma | MR Egger | 20 | 0.989(0.931 to 1.049) | 0.71 |
| 39S ribosomal protein L33, mitochondrial | prot-a-1942 | Lung adenocarcinoma | Weighted median | 20 | 0.995(0.947 to 1.046) | 0.853 |
| 39S ribosomal protein L33, mitochondrial | prot-a-1942 | Lung adenocarcinoma | Inverse variance weighted | 20 | 1.006(0.967 to 1.046) | 0.771 |
| Cytochrome c oxidase subunit 4 isoform 2, mitochondrial | prot-a-637 | Lung adenocarcinoma | MR Egger | 19 | 1.012(0.880 to 1.164) | 0.867 |
| Cytochrome c oxidase subunit 4 isoform 2, mitochondrial | prot-a-637 | Lung adenocarcinoma | Weighted median | 19 | 1.007(0.926 to 1.096) | 0.868 |
| Cytochrome c oxidase subunit 4 isoform 2, mitochondrial | prot-a-637 | Lung adenocarcinoma | Inverse variance weighted | 19 | 0.960(0.903 to 1.020) | 0.186 |
| Carbonic anhydrase 5A, mitochondrial | prot-a-332 | Lung adenocarcinoma | MR Egger | 14 | 1.046(0.858 to 1.275) | 0.665 |
| Carbonic anhydrase 5A, mitochondrial | prot-a-332 | Lung adenocarcinoma | Weighted median | 14 | 1.042(0.945 to 1.149) | 0.413 |
| Carbonic anhydrase 5A, mitochondrial | prot-a-332 | Lung adenocarcinoma | Inverse variance weighted | 14 | 1.040(0.967 to 1.118) | 0.296 |
| [Pyruvate dehydrogenase (acetyl-transferring)] kinase isozyme 1, mitochondrial | prot-a-2235 | Lung adenocarcinoma | MR Egger | 18 | 0.967(0.876 to 1.067) | 0.509 |
| [Pyruvate dehydrogenase (acetyl-transferring)] kinase isozyme 1, mitochondrial | prot-a-2235 | Lung adenocarcinoma | Weighted median | 18 | 0.994(0.933 to 1.058) | 0.843 |
| [Pyruvate dehydrogenase (acetyl-transferring)] kinase isozyme 1, mitochondrial | prot-a-2235 | Lung adenocarcinoma | Inverse variance weighted | 18 | 0.994(0.943 to 1.047) | 0.811 |
| Ribosome-recycling factor, mitochondrial | prot-a-1945 | Lung adenocarcinoma | MR Egger | 23 | 1.036(0.904 to 1.186) | 0.618 |
| Ribosome-recycling factor, mitochondrial | prot-a-1945 | Lung adenocarcinoma | Weighted median | 23 | 1.037(0.948 to 1.135) | 0.426 |
| Ribosome-recycling factor, mitochondrial | prot-a-1945 | Lung adenocarcinoma | Inverse variance weighted | 23 | 1.004(0.943 to 1.069) | 0.892 |
| ES1 protein homolog, mitochondrial | prot-a-308 | Lung adenocarcinoma | MR Egger | 22 | 0.897(0.765 to 1.053) | 0.199 |
| ES1 protein homolog, mitochondrial | prot-a-308 | Lung adenocarcinoma | Weighted median | 22 | 1.004(0.918 to 1.099) | 0.927 |
| ES1 protein homolog, mitochondrial | prot-a-308 | Lung adenocarcinoma | Inverse variance weighted | 22 | 1.027(0.963 to 1.095) | 0.421 |
| Dihydrolipoyl dehydrogenase, mitochondrial | prot-a-825 | Lung adenocarcinoma | MR Egger | 28 | 1.009(0.896 to 1.137) | 0.88 |
| Dihydrolipoyl dehydrogenase, mitochondrial | prot-a-825 | Lung adenocarcinoma | Weighted median | 28 | 1.001(0.927 to 1.081) | 0.983 |
| Dihydrolipoyl dehydrogenase, mitochondrial | prot-a-825 | Lung adenocarcinoma | Inverse variance weighted | 28 | 1.011(0.956 to 1.068) | 0.711 |
| GrpE protein homolog 1, mitochondrial | prot-a-1281 | Lung adenocarcinoma | MR Egger | 16 | 1.195(0.989 to 1.445) | 0.087 |
| GrpE protein homolog 1, mitochondrial | prot-a-1281 | Lung adenocarcinoma | Weighted median | 16 | 0.938(0.845 to 1.043) | 0.236 |
| GrpE protein homolog 1, mitochondrial | prot-a-1281 | Lung adenocarcinoma | Inverse variance weighted | 16 | 0.972(0.902 to 1.048) | 0.461 |
| Hydroxymethylglutaryl-CoA synthase, mitochondrial | prot-a-1356 | Lung adenocarcinoma | MR Egger | 17 | 0.965(0.792 to 1.176) | 0.732 |
| Hydroxymethylglutaryl-CoA synthase, mitochondrial | prot-a-1356 | Lung adenocarcinoma | Weighted median | 17 | 1.070(0.968 to 1.182) | 0.185 |
| Hydroxymethylglutaryl-CoA synthase, mitochondrial | prot-a-1356 | Lung adenocarcinoma | Inverse variance weighted | 17 | 1.057(0.982 to 1.139) | 0.141 |
| N-acetylglutamate synthase, mitochondrial | prot-a-1997 | Lung adenocarcinoma | MR Egger | 21 | 1.000(0.854 to 1.171) | 0.998 |
| N-acetylglutamate synthase, mitochondrial | prot-a-1997 | Lung adenocarcinoma | Weighted median | 21 | 1.026(0.936 to 1.124) | 0.584 |
| N-acetylglutamate synthase, mitochondrial | prot-a-1997 | Lung adenocarcinoma | Inverse variance weighted | 21 | 1.034(0.966 to 1.106) | 0.334 |
| Poly(A) RNA polymerase, mitochondrial | prot-a-1964 | Lung adenocarcinoma | MR Egger | 19 | 0.911(0.767 to 1.081) | 0.301 |
| Poly(A) RNA polymerase, mitochondrial | prot-a-1964 | Lung adenocarcinoma | Weighted median | 19 | 1.005(0.920 to 1.098) | 0.912 |
| Poly(A) RNA polymerase, mitochondrial | prot-a-1964 | Lung adenocarcinoma | Inverse variance weighted | 19 | 1.012(0.953 to 1.075) | 0.697 |
| 4-hydroxy-2-oxoglutarate aldolase, mitochondrial | prot-a-1368 | Lung adenocarcinoma | MR Egger | 7 | 1.011(0.740 to 1.380) | 0.949 |
| 4-hydroxy-2-oxoglutarate aldolase, mitochondrial | prot-a-1368 | Lung adenocarcinoma | Weighted median | 7 | 1.049(0.901 to 1.221) | 0.54 |
| 4-hydroxy-2-oxoglutarate aldolase, mitochondrial | prot-a-1368 | Lung adenocarcinoma | Inverse variance weighted | 7 | 0.997(0.882 to 1.128) | 0.964 |
| Pyruvate carboxylase, mitochondrial | prot-a-2190 | Lung adenocarcinoma | MR Egger | 19 | 1.018(0.844 to 1.228) | 0.856 |
| Pyruvate carboxylase, mitochondrial | prot-a-2190 | Lung adenocarcinoma | Weighted median | 19 | 0.975(0.886 to 1.074) | 0.61 |
| Pyruvate carboxylase, mitochondrial | prot-a-2190 | Lung adenocarcinoma | Inverse variance weighted | 19 | 0.971(0.898 to 1.050) | 0.466 |
| Phenylalanine--tRNA ligase, mitochondrial | prot-a-1055 | Lung adenocarcinoma | MR Egger | 14 | 0.988(0.855 to 1.142) | 0.874 |
| Phenylalanine--tRNA ligase, mitochondrial | prot-a-1055 | Lung adenocarcinoma | Weighted median | 14 | 0.984(0.896 to 1.081) | 0.734 |
| Phenylalanine--tRNA ligase, mitochondrial | prot-a-1055 | Lung adenocarcinoma | Inverse variance weighted | 14 | 0.970(0.904 to 1.040) | 0.385 |
| Cytochrome c oxidase subunit 7A1, mitochondrial | prot-a-640 | Lung adenocarcinoma | MR Egger | 27 | 0.936(0.826 to 1.060) | 0.306 |
| Cytochrome c oxidase subunit 7A1, mitochondrial | prot-a-640 | Lung adenocarcinoma | Weighted median | 27 | 1.003(0.926 to 1.087) | 0.936 |
| Cytochrome c oxidase subunit 7A1, mitochondrial | prot-a-640 | Lung adenocarcinoma | Inverse variance weighted | 27 | 0.982(0.929 to 1.039) | 0.536 |
| Diablo homolog, mitochondrial | prot-a-818 | Lung adenocarcinoma | MR Egger | 23 | 0.939(0.791 to 1.114) | 0.477 |
| Diablo homolog, mitochondrial | prot-a-818 | Lung adenocarcinoma | Weighted median | 23 | 0.964(0.882 to 1.053) | 0.417 |
| Diablo homolog, mitochondrial | prot-a-818 | Lung adenocarcinoma | Inverse variance weighted | 23 | 0.973(0.907 to 1.042) | 0.431 |
| Cytochrome c oxidase assembly factor 3 homolog, mitochondrial | prot-a-612 | Lung adenocarcinoma | MR Egger | 20 | 0.961(0.824 to 1.121) | 0.617 |
| Cytochrome c oxidase assembly factor 3 homolog, mitochondrial | prot-a-612 | Lung adenocarcinoma | Weighted median | 20 | 1.013(0.923 to 1.111) | 0.791 |
| Cytochrome c oxidase assembly factor 3 homolog, mitochondrial | prot-a-612 | Lung adenocarcinoma | Inverse variance weighted | 20 | 0.991(0.928 to 1.059) | 0.799 |
| Steroidogenic acute regulatory protein, mitochondrial | prot-a-2866 | Lung adenocarcinoma | MR Egger | 19 | 0.880(0.735 to 1.053) | 0.18 |
| Steroidogenic acute regulatory protein, mitochondrial | prot-a-2866 | Lung adenocarcinoma | Weighted median | 19 | 1.020(0.928 to 1.122) | 0.678 |
| Steroidogenic acute regulatory protein, mitochondrial | prot-a-2866 | Lung adenocarcinoma | Inverse variance weighted | 19 | 1.005(0.939 to 1.077) | 0.882 |
| Persulfide dioxygenase ETHE1, mitochondrial | prot-a-992 | Lung adenocarcinoma | MR Egger | 22 | 0.896(0.768 to 1.045) | 0.178 |
| Persulfide dioxygenase ETHE1, mitochondrial | prot-a-992 | Lung adenocarcinoma | Weighted median | 22 | 1.009(0.925 to 1.101) | 0.836 |
| Persulfide dioxygenase ETHE1, mitochondrial | prot-a-992 | Lung adenocarcinoma | Inverse variance weighted | 22 | 1.013(0.954 to 1.075) | 0.683 |
| Cytochrome c oxidase subunit 8A, mitochondrial | prot-a-641 | Lung adenocarcinoma | MR Egger | 16 | 1.033(0.945 to 1.129) | 0.488 |
| Cytochrome c oxidase subunit 8A, mitochondrial | prot-a-641 | Lung adenocarcinoma | Weighted median | 16 | 1.028(0.963 to 1.098) | 0.401 |
| Cytochrome c oxidase subunit 8A, mitochondrial | prot-a-641 | Lung adenocarcinoma | Inverse variance weighted | 16 | 0.996(0.940 to 1.056) | 0.901 |
| ADP-ribose pyrophosphatase, mitochondrial | prot-a-2129 | Lung adenocarcinoma | MR Egger | 20 | 0.909(0.836 to 0.990) | 0.042 |
| ADP-ribose pyrophosphatase, mitochondrial | prot-a-2129 | Lung adenocarcinoma | Weighted median | 20 | 0.933(0.869 to 1.001) | 0.053 |
| ADP-ribose pyrophosphatase, mitochondrial | prot-a-2129 | Lung adenocarcinoma | Inverse variance weighted | 20 | 0.968(0.922 to 1.016) | 0.191 |
| rRNA methyltransferase 3, mitochondrial | prot-a-2575 | Lung adenocarcinoma | MR Egger | 21 | 0.980(0.913 to 1.052) | 0.577 |
| rRNA methyltransferase 3, mitochondrial | prot-a-2575 | Lung adenocarcinoma | Weighted median | 21 | 0.999(0.953 to 1.048) | 0.966 |
| rRNA methyltransferase 3, mitochondrial | prot-a-2575 | Lung adenocarcinoma | Inverse variance weighted | 21 | 1.039(0.993 to 1.087) | 0.101 |
| Glutaredoxin-2, mitochondrial | prot-a-1220 | Lung adenocarcinoma | MR Egger | 25 | 0.953(0.862 to 1.055) | 0.365 |
| Glutaredoxin-2, mitochondrial | prot-a-1220 | Lung adenocarcinoma | Weighted median | 25 | 0.989(0.916 to 1.067) | 0.766 |
| Glutaredoxin-2, mitochondrial | prot-a-1220 | Lung adenocarcinoma | Inverse variance weighted | 25 | 1.003(0.950 to 1.060) | 0.909 |
| Leucine-rich PPR motif-containing protein, mitochondrial | prot-a-1783 | Lung adenocarcinoma | MR Egger | 21 | 1.047(0.977 to 1.123) | 0.206 |
| Leucine-rich PPR motif-containing protein, mitochondrial | prot-a-1783 | Lung adenocarcinoma | Weighted median | 21 | 1.027(0.975 to 1.081) | 0.315 |
| Leucine-rich PPR motif-containing protein, mitochondrial | prot-a-1783 | Lung adenocarcinoma | Inverse variance weighted | 21 | 1.005(0.964 to 1.048) | 0.819 |
| 39S ribosomal protein L32, mitochondrial | prot-a-1941 | Lung adenocarcinoma | MR Egger | 19 | 1.083(0.873 to 1.343) | 0.479 |
| 39S ribosomal protein L32, mitochondrial | prot-a-1941 | Lung adenocarcinoma | Weighted median | 19 | 1.012(0.922 to 1.112) | 0.795 |
| 39S ribosomal protein L32, mitochondrial | prot-a-1941 | Lung adenocarcinoma | Inverse variance weighted | 19 | 1.004(0.936 to 1.078) | 0.906 |
| Apoptosis-inducing factor 1, mitochondrial | prot-a-64 | Lung adenocarcinoma | MR Egger | 17 | 0.980(0.762 to 1.261) | 0.876 |
| Apoptosis-inducing factor 1, mitochondrial | prot-a-64 | Lung adenocarcinoma | Weighted median | 17 | 1.000(0.908 to 1.101) | 0.997 |
| Apoptosis-inducing factor 1, mitochondrial | prot-a-64 | Lung adenocarcinoma | Inverse variance weighted | 17 | 1.019(0.948 to 1.095) | 0.612 |
| Coiled-coil-helix-coiled-coil-helix domain-containing protein 10, mitochondrial | prot-a-534 | Lung adenocarcinoma | MR Egger | 21 | 1.004(0.907 to 1.111) | 0.947 |
| Coiled-coil-helix-coiled-coil-helix domain-containing protein 10, mitochondrial | prot-a-534 | Lung adenocarcinoma | Weighted median | 21 | 1.015(0.958 to 1.075) | 0.616 |
| Coiled-coil-helix-coiled-coil-helix domain-containing protein 10, mitochondrial | prot-a-534 | Lung adenocarcinoma | Inverse variance weighted | 21 | 1.022(0.962 to 1.086) | 0.473 |
| 39S ribosomal protein L34, mitochondrial | prot-a-1943 | Lung adenocarcinoma | MR Egger | 18 | 1.121(0.924 to 1.361) | 0.263 |
| 39S ribosomal protein L34, mitochondrial | prot-a-1943 | Lung adenocarcinoma | Weighted median | 18 | 1.005(0.915 to 1.104) | 0.917 |
| 39S ribosomal protein L34, mitochondrial | prot-a-1943 | Lung adenocarcinoma | Inverse variance weighted | 18 | 1.040(0.972 to 1.113) | 0.255 |
| Essential MCU regulator, mitochondrial | prot-a-2776 | Lung adenocarcinoma | MR Egger | 26 | 1.195(0.997 to 1.432) | 0.066 |
| Essential MCU regulator, mitochondrial | prot-a-2776 | Lung adenocarcinoma | Weighted median | 26 | 1.022(0.936 to 1.117) | 0.621 |
| Essential MCU regulator, mitochondrial | prot-a-2776 | Lung adenocarcinoma | Inverse variance weighted | 26 | 1.003(0.928 to 1.084) | 0.935 |
| Malonyl-CoA decarboxylase, mitochondrial | prot-a-1907 | Lung adenocarcinoma | MR Egger | 12 | 1.030(0.759 to 1.397) | 0.854 |
| Malonyl-CoA decarboxylase, mitochondrial | prot-a-1907 | Lung adenocarcinoma | Weighted median | 12 | 1.029(0.901 to 1.175) | 0.673 |
| Malonyl-CoA decarboxylase, mitochondrial | prot-a-1907 | Lung adenocarcinoma | Inverse variance weighted | 12 | 1.030(0.908 to 1.169) | 0.643 |
| Apoptosis-inducing factor 1, mitochondrial | prot-a-63 | Lung adenocarcinoma | MR Egger | 18 | 1.153(0.963 to 1.380) | 0.141 |
| Apoptosis-inducing factor 1, mitochondrial | prot-a-63 | Lung adenocarcinoma | Weighted median | 18 | 1.003(0.912 to 1.103) | 0.953 |
| Apoptosis-inducing factor 1, mitochondrial | prot-a-63 | Lung adenocarcinoma | Inverse variance weighted | 18 | 0.998(0.924 to 1.077) | 0.951 |
| Succinate dehydrogenase assembly factor 2, mitochondrial | prot-a-2657 | Lung adenocarcinoma | MR Egger | 19 | 1.055(0.887 to 1.255) | 0.552 |
| Succinate dehydrogenase assembly factor 2, mitochondrial | prot-a-2657 | Lung adenocarcinoma | Weighted median | 19 | 1.012(0.922 to 1.110) | 0.805 |
| Succinate dehydrogenase assembly factor 2, mitochondrial | prot-a-2657 | Lung adenocarcinoma | Inverse variance weighted | 19 | 1.005(0.939 to 1.076) | 0.892 |
| 39S ribosomal protein L52, mitochondrial | prot-a-1944 | Lung adenocarcinoma | MR Egger | 24 | 1.261(0.911 to 1.746) | 0.176 |
| 39S ribosomal protein L52, mitochondrial | prot-a-1944 | Lung adenocarcinoma | Weighted median | 24 | 0.969(0.891 to 1.054) | 0.466 |
| 39S ribosomal protein L52, mitochondrial | prot-a-1944 | Lung adenocarcinoma | Inverse variance weighted | 24 | 0.940(0.842 to 1.048) | 0.266 |
| NAD-dependent protein deacylase sirtuin-5, mitochondrial | prot-a-2737 | Lung adenocarcinoma | MR Egger | 21 | 0.929(0.772 to 1.117) | 0.44 |
| NAD-dependent protein deacylase sirtuin-5, mitochondrial | prot-a-2737 | Lung adenocarcinoma | Weighted median | 21 | 0.964(0.880 to 1.056) | 0.434 |
| NAD-dependent protein deacylase sirtuin-5, mitochondrial | prot-a-2737 | Lung adenocarcinoma | Inverse variance weighted | 21 | 0.968(0.907 to 1.033) | 0.323 |
| NADH dehydrogenase [ubiquinone] 1 beta subcomplex subunit 8, mitochondrial | prot-a-2024 | Lung adenocarcinoma | MR Egger | 18 | 0.971(0.797 to 1.184) | 0.777 |
| NADH dehydrogenase [ubiquinone] 1 beta subcomplex subunit 8, mitochondrial | prot-a-2024 | Lung adenocarcinoma | Weighted median | 18 | 0.938(0.847 to 1.039) | 0.219 |
| NADH dehydrogenase [ubiquinone] 1 beta subcomplex subunit 8, mitochondrial | prot-a-2024 | Lung adenocarcinoma | Inverse variance weighted | 18 | 0.970(0.898 to 1.047) | 0.429 |
| NADH dehydrogenase [ubiquinone] iron-sulfur protein 4, mitochondrial | prot-a-2025 | Lung adenocarcinoma | MR Egger | 29 | 0.985(0.942 to 1.030) | 0.507 |
| NADH dehydrogenase [ubiquinone] iron-sulfur protein 4, mitochondrial | prot-a-2025 | Lung adenocarcinoma | Weighted median | 29 | 0.985(0.950 to 1.022) | 0.423 |
| NADH dehydrogenase [ubiquinone] iron-sulfur protein 4, mitochondrial | prot-a-2025 | Lung adenocarcinoma | Inverse variance weighted | 29 | 0.981(0.951 to 1.013) | 0.244 |
| Protein SCO1 homolog, mitochondrial | prot-a-2653 | Lung adenocarcinoma | MR Egger | 22 | 0.875(0.718 to 1.066) | 0.199 |
| Protein SCO1 homolog, mitochondrial | prot-a-2653 | Lung adenocarcinoma | Weighted median | 22 | 1.012(0.931 to 1.101) | 0.776 |
| Protein SCO1 homolog, mitochondrial | prot-a-2653 | Lung adenocarcinoma | Inverse variance weighted | 22 | 1.003(0.943 to 1.066) | 0.93 |
| Superoxide dismutase [Mn], mitochondrial | prot-a-2799 | Lung adenocarcinoma | MR Egger | 22 | 0.984(0.900 to 1.077) | 0.735 |
| Superoxide dismutase [Mn], mitochondrial | prot-a-2799 | Lung adenocarcinoma | Weighted median | 22 | 0.950(0.898 to 1.005) | 0.076 |
| Superoxide dismutase [Mn], mitochondrial | prot-a-2799 | Lung adenocarcinoma | Inverse variance weighted | 22 | 0.979(0.930 to 1.030) | 0.413 |
| Transmembrane protein 70, mitochondrial | prot-a-3015 | Lung adenocarcinoma | MR Egger | 15 | 0.968(0.802 to 1.169) | 0.744 |
| Transmembrane protein 70, mitochondrial | prot-a-3015 | Lung adenocarcinoma | Weighted median | 15 | 1.053(0.953 to 1.163) | 0.312 |
| Transmembrane protein 70, mitochondrial | prot-a-3015 | Lung adenocarcinoma | Inverse variance weighted | 15 | 1.064(0.986 to 1.148) | 0.109 |
| Peptide chain release factor 1-like, mitochondrial | prot-a-1965 | Lung adenocarcinoma | MR Egger | 15 | 1.213(0.973 to 1.511) | 0.11 |
| Peptide chain release factor 1-like, mitochondrial | prot-a-1965 | Lung adenocarcinoma | Weighted median | 15 | 1.004(0.909 to 1.109) | 0.944 |
| Peptide chain release factor 1-like, mitochondrial | prot-a-1965 | Lung adenocarcinoma | Inverse variance weighted | 15 | 0.980(0.913 to 1.052) | 0.582 |
| Coiled-coil domain-containing protein 90B, mitochondrial | prot-a-385 | Lung adenocarcinoma | MR Egger | 16 | 1.000(0.826 to 1.212) | 0.998 |
| Coiled-coil domain-containing protein 90B, mitochondrial | prot-a-385 | Lung adenocarcinoma | Weighted median | 16 | 0.939(0.851 to 1.037) | 0.213 |
| Coiled-coil domain-containing protein 90B, mitochondrial | prot-a-385 | Lung adenocarcinoma | Inverse variance weighted | 16 | 0.949(0.882 to 1.022) | 0.166 |
| Calcium uptake protein 3, mitochondrial | prot-a-896 | Lung adenocarcinoma | MR Egger | 26 | 0.955(0.808 to 1.128) | 0.595 |
| Calcium uptake protein 3, mitochondrial | prot-a-896 | Lung adenocarcinoma | Weighted median | 26 | 1.000(0.922 to 1.084) | 0.998 |
| Calcium uptake protein 3, mitochondrial | prot-a-896 | Lung adenocarcinoma | Inverse variance weighted | 26 | 0.993(0.934 to 1.055) | 0.81 |
| tRNA pseudouridine synthase A, mitochondrial | prot-a-2454 | Lung adenocarcinoma | MR Egger | 17 | 1.021(0.820 to 1.272) | 0.854 |
| tRNA pseudouridine synthase A, mitochondrial | prot-a-2454 | Lung adenocarcinoma | Weighted median | 17 | 1.034(0.947 to 1.129) | 0.454 |
| tRNA pseudouridine synthase A, mitochondrial | prot-a-2454 | Lung adenocarcinoma | Inverse variance weighted | 17 | 1.025(0.956 to 1.098) | 0.485 |
| NADH dehydrogenase [ubiquinone] flavoprotein 2, mitochondrial | prot-a-2026 | Lung adenocarcinoma | MR Egger | 17 | 0.890(0.650 to 1.217) | 0.476 |
| NADH dehydrogenase [ubiquinone] flavoprotein 2, mitochondrial | prot-a-2026 | Lung adenocarcinoma | Weighted median | 17 | 0.922(0.818 to 1.038) | 0.179 |
| NADH dehydrogenase [ubiquinone] flavoprotein 2, mitochondrial | prot-a-2026 | Lung adenocarcinoma | Inverse variance weighted | 17 | 0.916(0.819 to 1.025) | 0.127 |
| Iron-sulfur cluster assembly enzyme ISCU, mitochondrial | prot-a-1572 | Lung adenocarcinoma | MR Egger | 19 | 1.122(0.929 to 1.354) | 0.248 |
| Iron-sulfur cluster assembly enzyme ISCU, mitochondrial | prot-a-1572 | Lung adenocarcinoma | Weighted median | 19 | 1.061(0.964 to 1.169) | 0.225 |
| Iron-sulfur cluster assembly enzyme ISCU, mitochondrial | prot-a-1572 | Lung adenocarcinoma | Inverse variance weighted | 19 | 1.052(0.984 to 1.126) | 0.138 |
| Complement component 1 Q subcomponent-binding protein, mitochondrial | prot-a-300 | Lung adenocarcinoma | MR Egger | 27 | 1.073(0.927 to 1.241) | 0.354 |
| Complement component 1 Q subcomponent-binding protein, mitochondrial | prot-a-300 | Lung adenocarcinoma | Weighted median | 27 | 1.023(0.947 to 1.105) | 0.566 |
| Complement component 1 Q subcomponent-binding protein, mitochondrial | prot-a-300 | Lung adenocarcinoma | Inverse variance weighted | 27 | 1.031(0.977 to 1.089) | 0.265 |
| Serine protease HTRA2, mitochondrial | prot-a-1392 | Lung adenocarcinoma | MR Egger | 15 | 1.125(0.905 to 1.399) | 0.309 |
| Serine protease HTRA2, mitochondrial | prot-a-1392 | Lung adenocarcinoma | Weighted median | 15 | 0.986(0.881 to 1.103) | 0.804 |
| Serine protease HTRA2, mitochondrial | prot-a-1392 | Lung adenocarcinoma | Inverse variance weighted | 15 | 0.995(0.906 to 1.092) | 0.91 |
| Cytochrome c oxidase subunit 5B, mitochondrial | prot-a-638 | Lung adenocarcinoma | MR Egger | 21 | 1.062(0.869 to 1.299) | 0.561 |
| Cytochrome c oxidase subunit 5B, mitochondrial | prot-a-638 | Lung adenocarcinoma | Weighted median | 21 | 0.936(0.861 to 1.016) | 0.115 |
| Cytochrome c oxidase subunit 5B, mitochondrial | prot-a-638 | Lung adenocarcinoma | Inverse variance weighted | 21 | 0.953(0.895 to 1.015) | 0.136 |
| [Pyruvate dehydrogenase (acetyl-transferring)] kinase isozyme 2, mitochondrial | prot-a-2236 | Lung adenocarcinoma | MR Egger | 15 | 1.070(0.856 to 1.336) | 0.562 |
| [Pyruvate dehydrogenase (acetyl-transferring)] kinase isozyme 2, mitochondrial | prot-a-2236 | Lung adenocarcinoma | Weighted median | 15 | 1.030(0.924 to 1.148) | 0.594 |
| [Pyruvate dehydrogenase (acetyl-transferring)] kinase isozyme 2, mitochondrial | prot-a-2236 | Lung adenocarcinoma | Inverse variance weighted | 15 | 1.082(0.996 to 1.175) | 0.064 |
| Lon protease homolog, mitochondrial | prot-a-1761 | Lung adenocarcinoma | MR Egger | 15 | 0.955(0.810 to 1.125) | 0.589 |
| Lon protease homolog, mitochondrial | prot-a-1761 | Lung adenocarcinoma | Weighted median | 15 | 1.000(0.909 to 1.099) | 0.995 |
| Lon protease homolog, mitochondrial | prot-a-1761 | Lung adenocarcinoma | Inverse variance weighted | 15 | 1.020(0.956 to 1.088) | 0.556 |
| Histidine triad nucleotide-binding protein 2, mitochondrial | prot-a-1339 | Lung adenocarcinoma | MR Egger | 18 | 0.985(0.820 to 1.183) | 0.874 |
| Histidine triad nucleotide-binding protein 2, mitochondrial | prot-a-1339 | Lung adenocarcinoma | Weighted median | 18 | 1.025(0.925 to 1.136) | 0.637 |
| Histidine triad nucleotide-binding protein 2, mitochondrial | prot-a-1339 | Lung adenocarcinoma | Inverse variance weighted | 18 | 1.024(0.950 to 1.103) | 0.538 |
| NADH dehydrogenase [ubiquinone] 1 beta subcomplex subunit 11, mitochondrial | prot-a-2022 | Lung adenocarcinoma | MR Egger | 21 | 0.961(0.819 to 1.129) | 0.635 |
| NADH dehydrogenase [ubiquinone] 1 beta subcomplex subunit 11, mitochondrial | prot-a-2022 | Lung adenocarcinoma | Weighted median | 21 | 0.996(0.910 to 1.091) | 0.935 |
| NADH dehydrogenase [ubiquinone] 1 beta subcomplex subunit 11, mitochondrial | prot-a-2022 | Lung adenocarcinoma | Inverse variance weighted | 21 | 1.011(0.950 to 1.077) | 0.728 |
| Serine--tRNA ligase, mitochondrial | prot-a-2627 | Lung adenocarcinoma | MR Egger | 22 | 0.949(0.759 to 1.186) | 0.651 |
| Serine--tRNA ligase, mitochondrial | prot-a-2627 | Lung adenocarcinoma | Weighted median | 22 | 1.008(0.920 to 1.104) | 0.872 |
| Serine--tRNA ligase, mitochondrial | prot-a-2627 | Lung adenocarcinoma | Inverse variance weighted | 22 | 1.000(0.927 to 1.080) | 0.991 |
| 39S ribosomal protein L14, mitochondrial | prot-a-1940 | Lung adenocarcinoma | MR Egger | 18 | 0.919(0.739 to 1.141) | 0.454 |
| 39S ribosomal protein L14, mitochondrial | prot-a-1940 | Lung adenocarcinoma | Weighted median | 18 | 1.031(0.932 to 1.141) | 0.556 |
| 39S ribosomal protein L14, mitochondrial | prot-a-1940 | Lung adenocarcinoma | Inverse variance weighted | 18 | 0.972(0.904 to 1.044) | 0.436 |
| Oligoribonuclease, mitochondrial | prot-a-2526 | Lung adenocarcinoma | MR Egger | 15 | 1.116(0.923 to 1.351) | 0.279 |
| Oligoribonuclease, mitochondrial | prot-a-2526 | Lung adenocarcinoma | Weighted median | 15 | 1.006(0.900 to 1.125) | 0.91 |
| Oligoribonuclease, mitochondrial | prot-a-2526 | Lung adenocarcinoma | Inverse variance weighted | 15 | 1.024(0.931 to 1.126) | 0.63 |
| Nucleoside diphosphate-linked moiety X motif 8, mitochondrial | prot-a-2128 | Lung adenocarcinoma | MR Egger | 21 | 0.962(0.823 to 1.126) | 0.638 |
| Nucleoside diphosphate-linked moiety X motif 8, mitochondrial | prot-a-2128 | Lung adenocarcinoma | Weighted median | 21 | 1.008(0.920 to 1.105) | 0.86 |
| Nucleoside diphosphate-linked moiety X motif 8, mitochondrial | prot-a-2128 | Lung adenocarcinoma | Inverse variance weighted | 21 | 0.969(0.909 to 1.032) | 0.328 |
| Methylmalonyl-CoA epimerase, mitochondrial | prot-a-1864 | Lung adenocarcinoma | MR Egger | 22 | 0.938(0.734 to 1.200) | 0.617 |
| Methylmalonyl-CoA epimerase, mitochondrial | prot-a-1864 | Lung adenocarcinoma | Weighted median | 22 | 1.027(0.930 to 1.135) | 0.597 |
| Methylmalonyl-CoA epimerase, mitochondrial | prot-a-1864 | Lung adenocarcinoma | Inverse variance weighted | 22 | 1.032(0.948 to 1.124) | 0.467 |
| ATP synthase subunit beta, mitochondrial | prot-a-203 | Lung adenocarcinoma | MR Egger | 24 | 1.021(0.887 to 1.174) | 0.779 |
| ATP synthase subunit beta, mitochondrial | prot-a-203 | Lung adenocarcinoma | Weighted median | 24 | 1.030(0.948 to 1.119) | 0.484 |
| ATP synthase subunit beta, mitochondrial | prot-a-203 | Lung adenocarcinoma | Inverse variance weighted | 24 | 1.005(0.948 to 1.067) | 0.857 |
| NFU1 iron-sulfur cluster scaffold homolog, mitochondrial | prot-a-2041 | Lung adenocarcinoma | MR Egger | 21 | 0.993(0.852 to 1.157) | 0.927 |
| NFU1 iron-sulfur cluster scaffold homolog, mitochondrial | prot-a-2041 | Lung adenocarcinoma | Weighted median | 21 | 1.059(0.967 to 1.161) | 0.217 |
| NFU1 iron-sulfur cluster scaffold homolog, mitochondrial | prot-a-2041 | Lung adenocarcinoma | Inverse variance weighted | 21 | 1.051(0.982 to 1.124) | 0.149 |
| Mitochondrial ubiquitin ligase activator of NFKB 1 | prot-a-1970 | Lung adenocarcinoma | MR Egger | 22 | 1.022(0.889 to 1.175) | 0.766 |
| Mitochondrial ubiquitin ligase activator of NFKB 1 | prot-a-1970 | Lung adenocarcinoma | Weighted median | 22 | 0.962(0.880 to 1.052) | 0.397 |
| Mitochondrial ubiquitin ligase activator of NFKB 1 | prot-a-1970 | Lung adenocarcinoma | Inverse variance weighted | 22 | 0.984(0.924 to 1.048) | 0.624 |
| Mitochondrial fission regulator 1 | prot-a-1961 | Lung adenocarcinoma | MR Egger | 13 | 0.915(0.772 to 1.084) | 0.327 |
| Mitochondrial fission regulator 1 | prot-a-1961 | Lung adenocarcinoma | Weighted median | 13 | 0.936(0.840 to 1.043) | 0.229 |
| Mitochondrial fission regulator 1 | prot-a-1961 | Lung adenocarcinoma | Inverse variance weighted | 13 | 0.982(0.910 to 1.059) | 0.634 |
| Mitochondrial glutamate carrier 2 | prot-a-2749 | Lung adenocarcinoma | MR Egger | 19 | 0.986(0.817 to 1.190) | 0.884 |
| Mitochondrial glutamate carrier 2 | prot-a-2749 | Lung adenocarcinoma | Weighted median | 19 | 0.994(0.899 to 1.099) | 0.91 |
| Mitochondrial glutamate carrier 2 | prot-a-2749 | Lung adenocarcinoma | Inverse variance weighted | 19 | 1.009(0.936 to 1.089) | 0.81 |
| Mitochondrial peptide methionine sulfoxide reductase | prot-a-1953 | Lung adenocarcinoma | MR Egger | 20 | 0.845(0.725 to 0.985) | 0.045 |
| Mitochondrial peptide methionine sulfoxide reductase | prot-a-1953 | Lung adenocarcinoma | Weighted median | 20 | 1.003(0.909 to 1.106) | 0.954 |
| Mitochondrial peptide methionine sulfoxide reductase | prot-a-1953 | Lung adenocarcinoma | Inverse variance weighted | 20 | 0.974(0.907 to 1.047) | 0.474 |
| Mitochondrial sodium/hydrogen exchanger 9B2 | prot-a-2764 | Lung adenocarcinoma | MR Egger | 24 | 1.005(0.841 to 1.201) | 0.956 |
| Mitochondrial sodium/hydrogen exchanger 9B2 | prot-a-2764 | Lung adenocarcinoma | Weighted median | 24 | 0.966(0.885 to 1.054) | 0.433 |
| Mitochondrial sodium/hydrogen exchanger 9B2 | prot-a-2764 | Lung adenocarcinoma | Inverse variance weighted | 24 | 0.971(0.909 to 1.037) | 0.383 |
| Mitochondrial import inner membrane translocase subunit TIM14 | prot-a-847 | Lung adenocarcinoma | MR Egger | 19 | 0.906(0.761 to 1.079) | 0.284 |
| Mitochondrial import inner membrane translocase subunit TIM14 | prot-a-847 | Lung adenocarcinoma | Weighted median | 19 | 0.970(0.882 to 1.067) | 0.535 |
| Mitochondrial import inner membrane translocase subunit TIM14 | prot-a-847 | Lung adenocarcinoma | Inverse variance weighted | 19 | 0.966(0.902 to 1.033) | 0.311 |
| Mitochondrial ubiquitin ligase activator of NFKB 1 | prot-a-1969 | Lung adenocarcinoma | MR Egger | 19 | 1.064(0.910 to 1.246) | 0.446 |
| Mitochondrial ubiquitin ligase activator of NFKB 1 | prot-a-1969 | Lung adenocarcinoma | Weighted median | 19 | 0.982(0.901 to 1.071) | 0.683 |
| Mitochondrial ubiquitin ligase activator of NFKB 1 | prot-a-1969 | Lung adenocarcinoma | Inverse variance weighted | 19 | 1.007(0.945 to 1.074) | 0.8240 |
| SNP: single nucleotide polymorphism. | | | | | | |

| Table S3. Characteristics of GWAS-identified different pathological types of LC-associated genetic instruments. | | | | | | | | | | |
| --- | --- | --- | --- | --- | --- | --- | --- | --- | --- | --- |
| **Expsure** | **SNP** | **Chromosome** | **Position** | **Effect allele** | **Other allele** | **Effect allele frequency** | **Beta** | **Standard Error** | ***P*-value** | ***F*-statistic** |
| LC | rs10265693 | 7 | 130720805 | G | A | 0.086134 | 0.09404 | 0.0197 | 0.00000187 | 22.72903952 |
|  | rs10811609 | 9 | 21746358 | G | A | 0.093085 | 0.094485 | 0.0197 | 0.00000161 | 23.01694787 |
|  | rs10904377 | 10 | 4978419 | T | G | 0.049066 | 0.134416 | 0.0284 | 0.00000224 | 22.37514782 |
|  | rs10984970 | 9 | 123400421 | A | G | 0.07128 | -0.107746 | 0.0231 | 0.0000032 | 21.69343193 |
|  | rs11032090 | 11 | 4417129 | G | A | 0.196298 | 0.067005 | 0.0150 | 0.00000753 | 20.05242671 |
|  | rs111308888 | 2 | 238071157 | C | T | 0.037099 | -0.152158 | 0.0340 | 0.00000746 | 20.07092364 |
|  | rs112813285 | 20 | 62537170 | T | C | 0.069826 | -0.120372 | 0.0235 | 0.000000297 | 26.26997687 |
|  | rs113659074 | 4 | 100252308 | T | G | 0.066888 | -0.106444 | 0.0225 | 0.00000222 | 22.39629037 |
|  | rs11697662 | 20 | 61992005 | T | C | 0.802326 | -0.075579 | 0.0157 | 0.00000158 | 23.05300325 |
|  | rs117534741 | 6 | 72384541 | A | G | 0.024213 | 0.211216 | 0.0419 | 0.000000468 | 25.39123533 |
|  | rs11780471 | 8 | 27344719 | A | G | 0.059619 | -0.141138 | 0.0250 | 1.69E-08 | 31.81767626 |
|  | rs12081674 | 1 | 90337025 | G | A | 0.184061 | 0.070788 | 0.0154 | 0.00000414 | 21.19997738 |
|  | rs13054277 | 22 | 23626896 | A | G | 0.238789 | 0.061913 | 0.0140 | 0.00000953 | 19.60156435 |
|  | rs13080835 | 3 | 189357199 | T | G | 0.492888 | -0.056779 | 0.0117 | 0.00000125 | 23.49388864 |
|  | rs148791337 | 7 | 86986144 | T | C | 0.022993 | -0.283516 | 0.0583 | 0.00000116 | 23.64954566 |
|  | rs150211 | 21 | 19415773 | C | T | 0.038294 | -0.151247 | 0.0326 | 0.00000359 | 21.47285128 |
|  | rs151118057 | 15 | 78988620 | A | G | 0.017665 | -0.293875 | 0.0512 | 9.56E-09 | 32.92719356 |
|  | rs1629083 | 11 | 118126576 | T | C | 0.502439 | -0.067017 | 0.0118 | 1.25E-08 | 32.40852946 |
|  | rs17181550 | 17 | 70299958 | G | T | 0.426501 | -0.062015 | 0.0119 | 0.000000198 | 27.04826595 |
|  | rs1866631 | 2 | 174075761 | A | G | 0.602312 | 0.058939 | 0.0119 | 0.000000697 | 24.62117362 |
|  | rs188586526 | 8 | 55495833 | T | G | 0.011967 | 0.269106 | 0.0564 | 0.00000183 | 22.76072137 |
|  | rs191205566 | 9 | 102587233 | T | C | 0.015135 | 0.337718 | 0.0637 | 0.000000117 | 28.07557604 |
|  | rs1991625 | 2 | 153831482 | G | A | 0.322123 | -0.057781 | 0.0125 | 0.00000399 | 21.26802468 |
|  | rs215335 | 12 | 47729595 | C | T | 0.510634 | -0.05488 | 0.0120 | 0.00000441 | 21.07969064 |
|  | rs2277866 | 20 | 19701943 | C | A | 0.79748 | 0.070132 | 0.0148 | 0.00000203 | 22.57001838 |
|  | rs2289393 | 2 | 24928579 | C | T | 0.267427 | 0.062293 | 0.0132 | 0.00000248 | 22.18254256 |
|  | rs2404618 | 8 | 1462268 | T | C | 0.465822 | 0.05306 | 0.0118 | 0.00000651 | 20.33259691 |
|  | rs3094604 | 6 | 31434111 | G | A | 0.16117 | 0.13992 | 0.0157 | 5.29E-19 | 79.31252973 |
|  | rs34357052 | 5 | 141195546 | C | T | 0.292529 | 0.060623 | 0.0130 | 0.000003 | 21.8197201 |
|  | rs367645 | 6 | 33024499 | A | G | 0.030624 | -0.175757 | 0.0364 | 0.00000136 | 23.33811975 |
|  | rs3847711 | 12 | 58834035 | G | A | 0.127131 | 0.090943 | 0.0178 | 0.00000034 | 26.00927871 |
|  | rs4722166 | 7 | 22738762 | A | C | 0.627022 | -0.05582 | 0.0124 | 0.00000648 | 20.33942864 |
|  | rs4774488 | 15 | 47491291 | C | T | 0.404215 | 0.06581 | 0.0119 | 0.000000036 | 30.3529803 |
|  | rs55902839 | 2 | 202505989 | C | A | 0.526515 | -0.056526 | 0.0126 | 0.00000744 | 20.07442037 |
|  | rs56377826 | 15 | 101827491 | C | T | 0.219257 | -0.070479 | 0.0155 | 0.00000558 | 20.62708405 |
|  | rs62396221 | 6 | 26285748 | A | C | 0.011112 | -0.279 | 0.0604 | 0.0000039 | 21.31255871 |
|  | rs62560775 | 9 | 22052068 | G | A | 0.103628 | 0.100389 | 0.0201 | 0.000000602 | 24.90455975 |
|  | rs631644 | 18 | 2280388 | G | A | 0.404002 | -0.064977 | 0.0139 | 0.00000306 | 21.7792807 |
|  | rs6989042 | 8 | 40182303 | G | A | 0.196347 | 0.07311 | 0.0163 | 0.00000695 | 20.2063887 |
|  | rs71519637 | 8 | 27334098 | T | C | 0.103324 | 0.103345 | 0.0202 | 0.000000312 | 26.17375705 |
|  | rs72949976 | 2 | 214034104 | T | C | 0.381966 | 0.055498 | 0.0121 | 0.0000046 | 20.99831873 |
|  | rs76486688 | 3 | 139776799 | A | G | 0.015709 | 0.230905 | 0.0513 | 0.00000673 | 20.26707818 |
|  | rs7683574 | 4 | 9719148 | T | C | 0.354685 | 0.059086 | 0.0128 | 0.00000405 | 21.24139311 |
|  | rs77045810 | 1 | 168505017 | C | A | 0.116771 | -0.098034 | 0.0187 | 0.000000151 | 27.56818376 |
|  | rs77468143 | 15 | 49376624 | G | T | 0.253876 | -0.082663 | 0.0135 | 0.000000001 | 37.31540964 |
|  | rs78062588 | 1 | 154566225 | C | T | 0.058231 | -0.1246 | 0.0247 | 0.00000046 | 25.42408371 |
|  | rs78334599 | 11 | 115998756 | A | G | 0.036319 | -0.155896 | 0.0316 | 0.000000793 | 24.37351004 |
|  | rs78853063 | 11 | 57250026 | T | C | 0.07529 | -0.118183 | 0.0234 | 0.000000465 | 25.40319113 |
|  | rs79368540 | 2 | 45189737 | T | C | 0.149195 | 0.088783 | 0.0178 | 0.000000613 | 24.87206848 |
|  | rs9869622 | 3 | 16671821 | T | C | 0.180788 | -0.072601 | 0.0152 | 0.00000173 | 22.86741628 |
| LUSC | rs10146472 | 14 | 34015021 | G | A | 0.197674 | -0.11818 | 0.0232 | 0.000000357 | 25.91414112 |
|  | rs10174077 | 2 | 152481712 | T | C | 0.476669 | 0.094664 | 0.0196 | 0.00000133 | 23.37147879 |
|  | rs111960002 | 8 | 144722420 | C | T | 0.045639 | 0.310149 | 0.0615 | 0.000000462 | 25.41524737 |
|  | rs113211131 | 7 | 22069637 | T | G | 0.098953 | -0.153627 | 0.0338 | 0.00000535 | 20.70573827 |
|  | rs11571818 | 13 | 32968810 | C | T | 0.010732 | 0.753896 | 0.0939 | 9.77E-16 | 64.4732667 |
|  | rs116238841 | 2 | 226622221 | A | G | 0.030547 | 0.283598 | 0.0629 | 0.00000642 | 20.35890183 |
|  | rs11697662 | 20 | 61992005 | T | C | 0.807235 | -0.113843 | 0.0251 | 0.0000055 | 20.65301343 |
|  | rs117194419 | 17 | 39570718 | A | G | 0.025369 | -0.350983 | 0.0791 | 0.00000915 | 19.68070823 |
|  | rs117534741 | 6 | 72384541 | A | G | 0.024812 | 0.341053 | 0.0683 | 0.000000599 | 24.91556223 |
|  | rs117723999 | 7 | 97097419 | T | G | 0.015538 | 0.450515 | 0.0981 | 0.00000441 | 21.07619879 |
|  | rs118166185 | 13 | 112103902 | T | G | 0.043662 | -0.199486 | 0.0435 | 0.00000462 | 20.98719636 |
|  | rs12957236 | 18 | 41899245 | T | G | 0.252375 | 0.095798 | 0.0214 | 0.00000782 | 19.97899819 |
|  | rs13031455 | 2 | 17784157 | C | T | 0.404765 | -0.089861 | 0.0190 | 0.00000223 | 22.38891519 |
|  | rs138809958 | 10 | 72030898 | C | A | 0.01719 | 0.359268 | 0.0751 | 0.00000175 | 22.85659645 |
|  | rs144279132 | 5 | 46198448 | G | A | 0.060975 | 0.251169 | 0.0559 | 0.00000714 | 20.15632103 |
|  | rs148373424 | 20 | 2497059 | T | G | 0.01114 | 0.455969 | 0.1020 | 0.00000785 | 19.97301609 |
|  | rs1534979 | 20 | 2331513 | C | T | 0.675833 | -0.090793 | 0.0199 | 0.0000049 | 20.87620295 |
|  | rs17811625 | 7 | 51284215 | T | C | 0.0401 | -0.247793 | 0.0551 | 0.00000687 | 20.2273932 |
|  | rs184716813 | 9 | 114151247 | C | T | 0.019177 | 0.329811 | 0.0720 | 0.00000463 | 20.98222327 |
|  | rs252152 | 5 | 141445774 | G | A | 0.65366 | -0.089705 | 0.0197 | 0.00000552 | 20.64815389 |
|  | rs28372851 | 10 | 101946033 | A | G | 0.042268 | 0.222015 | 0.0442 | 0.000000518 | 25.19515189 |
|  | rs28745309 | 5 | 82418056 | T | G | 0.010076 | 0.402415 | 0.0884 | 0.00000533 | 20.71254783 |
|  | rs3025388 | 9 | 136503256 | G | A | 0.17472 | -0.119232 | 0.0254 | 0.0000026 | 22.09367218 |
|  | rs3094604 | 6 | 31434111 | G | A | 0.1551 | 0.223155 | 0.0250 | 3.83E-19 | 79.95571558 |
|  | rs34783982 | 15 | 89515163 | A | G | 0.294931 | -0.092194 | 0.0208 | 0.00000912 | 19.68720468 |
|  | rs35028925 | 15 | 43930606 | C | A | 0.440061 | -0.094583 | 0.0200 | 0.00000224 | 22.37309867 |
|  | rs3754287 | 1 | 41952597 | T | C | 0.142572 | 0.129897 | 0.0271 | 0.00000166 | 22.95415737 |
|  | rs4453114 | 10 | 4961021 | C | T | 0.050834 | 0.207186 | 0.0436 | 0.00000204 | 22.55568738 |
|  | rs56319043 | 1 | 44171211 | T | C | 0.252121 | -0.099632 | 0.0216 | 0.00000393 | 21.29899454 |
|  | rs6957511 | 7 | 130668618 | T | C | 0.602151 | -0.095069 | 0.0194 | 0.000000978 | 23.96928516 |
|  | rs72949976 | 2 | 214034104 | T | C | 0.37741 | 0.088371 | 0.0194 | 0.00000521 | 20.755664 |
|  | rs75685923 | 9 | 136275229 | T | C | 0.032197 | 0.325454 | 0.0653 | 0.000000616 | 24.85983857 |
|  | rs7591446 | 2 | 45834076 | C | T | 0.809729 | 0.107478 | 0.0235 | 0.00000473 | 20.94147481 |
|  | rs7658584 | 4 | 89096641 | A | G | 0.154582 | 0.119432 | 0.0253 | 0.00000241 | 22.23617757 |
|  | rs77710113 | 1 | 154618623 | C | T | 0.058033 | -0.177102 | 0.0391 | 0.0000058 | 20.5542658 |
|  | rs78276179 | 11 | 2021493 | C | T | 0.089914 | 0.171514 | 0.0377 | 0.00000537 | 20.70116742 |
|  | rs78359677 | 1 | 90372770 | G | A | 0.229878 | 0.112066 | 0.0233 | 0.00000146 | 23.19415348 |
|  | rs78663305 | 1 | 168497741 | G | A | 0.117951 | -0.139422 | 0.0299 | 0.0000031 | 21.75398846 |
|  | rs9544837 | 13 | 79105039 | A | G | 0.515283 | -0.089798 | 0.0190 | 0.00000223 | 22.38581451 |
| SCLC | rs10118776 | 9 | 6227418 | A | G | 0.937186 | -0.291922 | 0.0575 | 0.000000391 | 25.73610446 |
|  | rs116039120 | 14 | 105103758 | C | T | 0.019935 | 0.476146 | 0.1046 | 0.00000535 | 20.7057179 |
|  | rs116877011 | 7 | 12653059 | T | C | 0.0204 | 0.515075 | 0.1092 | 0.00000241 | 22.23254794 |
|  | rs117205897 | 7 | 27019791 | T | C | 0.017012 | 0.62167 | 0.1386 | 0.00000727 | 20.11963048 |
|  | rs117490868 | 13 | 54915579 | A | C | 0.029304 | 0.538986 | 0.1124 | 0.00000163 | 22.99046095 |
|  | rs117729306 | 11 | 8487170 | C | T | 0.01066 | 0.836378 | 0.1683 | 0.000000672 | 24.69101876 |
|  | rs117823901 | 13 | 69847018 | C | A | 0.028044 | 0.414181 | 0.0900 | 0.00000421 | 21.16498934 |
|  | rs12279741 | 11 | 94284529 | G | A | 0.013704 | 0.601143 | 0.1346 | 0.00000791 | 19.95813137 |
|  | rs12981718 | 19 | 54567858 | A | G | 0.071553 | 0.307185 | 0.0592 | 0.000000213 | 26.90827417 |
|  | rs140013431 | 6 | 7770511 | C | T | 0.021231 | 0.508884 | 0.1045 | 0.00000111 | 23.72793504 |
|  | rs140394255 | 17 | 4327983 | A | G | 0.075062 | 0.266227 | 0.0562 | 0.0000022 | 22.40588475 |
|  | rs140641467 | 4 | 173779858 | A | C | 0.014927 | 0.657457 | 0.1442 | 0.00000512 | 20.78961588 |
|  | rs145474218 | 15 | 97982134 | C | T | 0.012957 | 0.673428 | 0.1447 | 0.00000324 | 21.6680269 |
|  | rs1703426 | 5 | 133185535 | T | C | 0.174936 | -0.186145 | 0.0388 | 0.00000159 | 23.03120885 |
|  | rs2586086 | 17 | 56016830 | G | A | 0.974242 | -0.56515 | 0.1226 | 0.000004 | 21.26462432 |
|  | rs258892 | 5 | 72305846 | C | A | 0.139422 | 0.188362 | 0.0413 | 0.00000501 | 20.8336713 |
|  | rs2704201 | 15 | 58418128 | C | T | 0.075227 | 0.250795 | 0.0545 | 0.00000421 | 21.16573814 |
|  | rs3134425 | 11 | 122709178 | C | T | 0.619024 | 0.149727 | 0.0311 | 0.00000147 | 23.18819251 |
|  | rs34735495 | 11 | 90410142 | G | A | 0.034076 | 0.383918 | 0.0835 | 0.00000432 | 21.11644401 |
|  | rs55656647 | 4 | 181959788 | C | T | 0.087723 | 0.271255 | 0.0602 | 0.00000667 | 20.28322518 |
|  | rs55843245 | 8 | 13437876 | A | C | 0.326033 | 0.148767 | 0.0319 | 0.00000309 | 21.7563699 |
|  | rs6937371 | 6 | 108322124 | A | G | 0.44779 | -0.165947 | 0.0369 | 0.00000669 | 20.27702449 |
|  | rs74501188 | 5 | 90226979 | A | G | 0.223943 | -0.183821 | 0.0359 | 0.000000298 | 26.26126729 |
|  | rs77028233 | 12 | 91031636 | A | G | 0.05861 | 0.314398 | 0.0706 | 0.00000835 | 19.85491016 |
|  | rs77216150 | 14 | 89667917 | T | C | 0.029214 | 0.434176 | 0.0961 | 0.00000626 | 20.40432782 |
|  | rs80152782 | 3 | 107247430 | A | G | 0.014092 | 0.56852 | 0.1283 | 0.00000944 | 19.61993461 |
|  | rs8049634 | 16 | 84225679 | G | A | 0.248146 | 0.164882 | 0.0341 | 0.00000137 | 23.32431359 |
|  | rs847245 | 14 | 72716686 | A | G | 0.9589 | -0.413767 | 0.0875 | 0.00000228 | 22.33945163 |
|  | rs9308062 | 4 | 164438200 | C | T | 0.583469 | -0.152783 | 0.0305 | 0.000000547 | 25.09079127 |
|  | rs9464379 | 6 | 56203674 | T | G | 0.416734 | -0.147816 | 0.0332 | 0.00000846 | 19.82840047 |
| F-statistic was calculated using the following formulas: F=R^2 (n-2)⁄(1-R^2) and R^2=2×MAF×(1-MAF)×β^2, where F represents F-statistic, R^2 represents the phenotypic variance explained by a genetic instrument, N is the sample size, β is the estimated genetic association of SNP with the exposure. SNP: single nucleotide polymorphism. LC, lung cancer. LUAD, lung adenocarcinoma. LUSC, lung squamous cell carcinoma. SCLC, small cell lung carcinoma. | | | | | | | | | | |

**Figure S1**


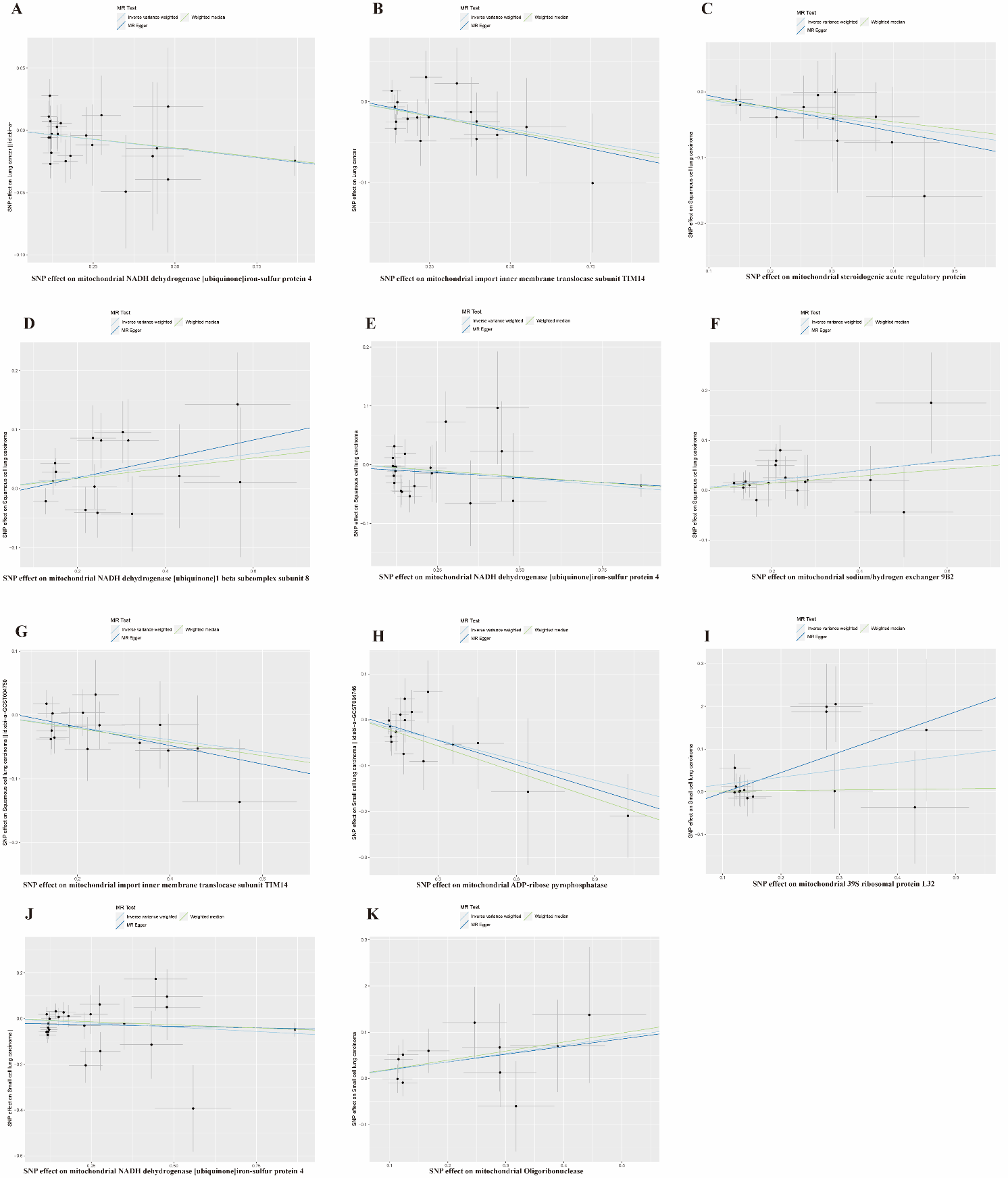


Figure
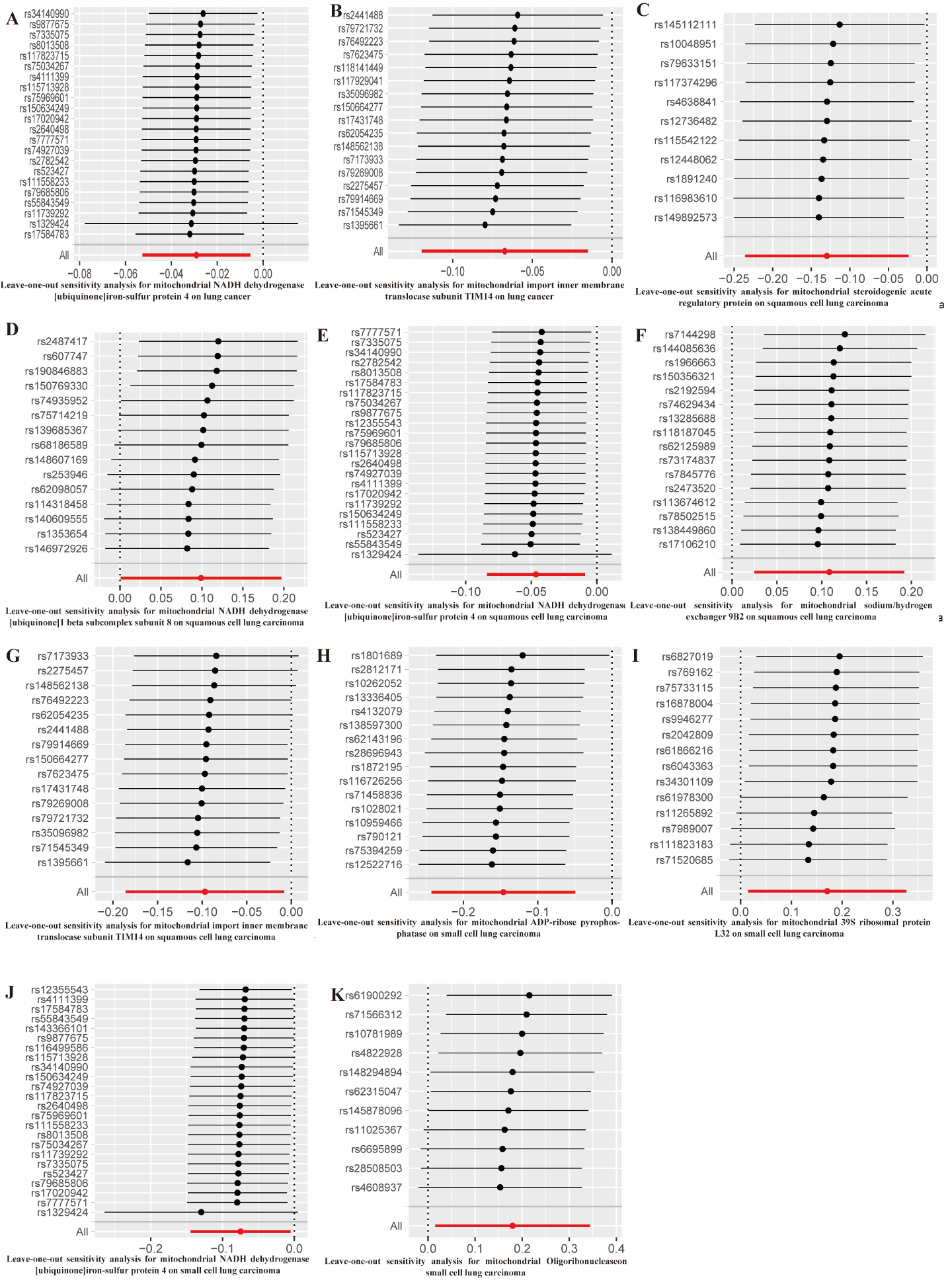
S2
